# Bridging Mechanisms and Strategies: MXene-Based Electrocatalysts for the Oxygen Evolution Reaction

**DOI:** 10.3390/nano16150947

**Published:** 2026-07-31

**Authors:** Hanzihou Zou, Ying Guo, Ting Yang, Honglin Gao

**Affiliations:** 1 Marine Engineering College, Dalian Maritime University, Dalian 116026, China; 2 Hangzhou Dahua Apparatus Manufacture Co., Ltd., Hangzhou 311400, China; 3 College of Transportation Engineering, Dalian Maritime University, Dalian 116026, China

**Keywords:** MXenes, oxygen evolution reaction, adsorbate evolution mechanism, lattice oxygen mechanism, interface engineering, morphology engineering, atomic-level regulation, oxidative stability

## Abstract

The oxygen evolution reaction (OER) is a key kinetic bottleneck in water electrolysis because it involves multistep proton-coupled electron transfer, the evolution of oxygen-containing intermediates and O–O bond formation. MXenes, as two-dimensional transition-metal carbides, nitrides and carbonitrides, possess high electrical conductivity, hydrophilic surfaces, tunable surface terminations and adjustable layered structures, making them promising platforms for OER catalyst design. However, their limited intrinsic active sites, sheet restacking and oxidative instability under anodic conditions restrict their direct application. This review firstly discusses the fundamental OER pathways based on the adsorbate evolution mechanism (AEM), lattice oxygen mechanism (LOM) and oxide path mechanism (OPM), providing a mechanistic basis for understanding intermediate adsorption, oxygen activation and working-state evolution. Then, a system framework from low-dimensional and micro-level control to high-dimensional and macro-level integration is constructed. The framework covers four levels: atom and local structure, interface, morphology and composite electrode. Drawing on specific examples, this review analyzes the characteristics and mechanisms of modification strategies from four different perspectives, starting with the basic principles of modification. These strategies include micro-scale, low-dimensional approaches such as “Vacancy and other atomic-Level Regulation”, macro-scale, high-dimensional methods like “Composite Engineering”, as well as intermediate approaches involving “Interface engineering” and “morphology engineering”. Special emphasis is placed on distinguishing between beneficial surface reconstruction of catalytically active hydroxyl oxide species and destructive oxidation. Finally, the review identified the unresolved key challenges, including the fuzziness of active sites, the diversity of initial material states and the lack of stability under industrial conditions, and looked forward to the future direction of reasonable design, operational characterization and device-level evaluation. Through this cross-scale analysis, this review aims to clarify the relationship between structure–activity–stability, and provide practical guidance for designing efficient, durable and experimentally verifiable MXene-based OER electrodes.

## 1. Introduction

With the accelerated transition of the global energy system and the continuously increasing share of renewable electricity, converting electrical energy into chemical fuels that can be stored, transported and readily utilized has become a central issue in energy research. Water electrolysis driven by renewable electricity is widely regarded as a key route for green hydrogen production because it relies on abundant feedstocks, delivers high-purity hydrogen and can be directly coupled with intermittent power sources such as wind and solar energy [1,2,3].

Water electrolysis consists of two half-reactions, namely the cathodic hydrogen evolution reaction (HER) and the anodic OER. At the cathode, H_2_O accepts electrons to produce hydrogen: 2H_2_O + 2e^−^ → H_2_ + 2OH^−^, whereas at the anode, OH^−^ loses electrons to generate oxygen: 4OH^−^ → O_2_ + 2H_2_O + 4e^−^. During HER, the reaction in acidic media usually begins with the Volmer step involving proton adsorption, followed by either electrochemical desorption via the Heyrovsky step or chemical recombination via the Tafel step to produce H_2_. In alkaline media, however, the dissociation of H_2_O is additionally needed to provide adsorbed hydrogen; therefore, the activation of water molecules often constitutes an additional kinetic limitation. By contrast, OER usually involves the formation and conversion of oxygen-containing intermediates such as *OH, *O and *OOH, where * denotes an active surface site, together with O–O bond formation and, in many transition-metal-based catalysts, surface reconstruction. Moreover, in some systems, the reaction pathway may also involve the direct participation of lattice oxygen. HER at the cathode is usually kinetically faster, whereas OER at the anode involves a multistep proton-coupled electron transfer process, continuous conversion of surface intermediates and more complex bond formation and cleavage; therefore, it often dominates the additional voltage loss and kinetic bottleneck of the overall water splitting [4,5,6]. The understanding of OER has evolved from early descriptions centered on surface-adsorbed intermediates to two key frameworks represented by the AEM and LOM. While in situ characterization techniques have been progressively introduced to track the actual reaction pathway and working-state evolution, some novel OER mechanisms represented by OPM have also been proposed continuously [6,7]. Nowadays, operando characterization has been indispensable for identifying the real active sites of MXene-based OER catalysts and tracking their structural evolution under working conditions. Techniques such as operando Raman spectroscopy, X-ray absorption spectroscopy (XAS), X-ray diffraction (XRD) and ambient-pressure X-ray photoelectron spectroscopy (XPS) can follow changes in oxidation state, local coordination, interfacial bonding, phase composition and surface terminations as the applied potential increases. These measurements can distinguish the original MXene phase from reconstructed oxyhydroxides and determine whether MXene functions as an active site, conductive support, precursor or interfacial regulator. When combined with isotope labeling, oxygen quantification and post-reaction microscopy, operando evidence can support more reliable mechanistic analysis. Such understanding is critical for establishing working-state structure–activity relationships and guiding the rational design of active and anodically durable MXene-based OER catalysts.

The development of OER catalysts that combine high activity, high stability and low cost has always remained at the core of water electrolysis research. RuO_2_ and IrO_2_ are still the most representative benchmark catalysts, but these noble-metal catalysts face some practical problems such as high cost, resource constraints and insufficient stability, making them difficult to deploy directly in large-scale applications [8,9]. Non-noble-metal catalysts have therefore attracted increasing attention because of their low cost, abundant elemental reserves and greater potential for large-scale application [10,11]. Nevertheless, these systems often still suffer from limited intrinsic activity, sluggish charge-transfer kinetics and insufficient structural stability under OER conditions, making it difficult to simultaneously achieve high activity and long-term durability [10,11]. Their active sites, interfacial charge-transfer behavior and working-state evolution are also often more complex and less controllable than those of benchmark noble-metal catalysts. Therefore, compositional, interfacial, structural and working-state regulation of non-noble-metal systems has become an essential route toward improving the economic viability and sustainability of water electrolysis. MXenes provide a new materials platform for addressing this issue. Since Naguib et al. first obtained Ti_3_C_2_ two-dimensional nanocrystals in 2011 through the selective etching of MAX phases, MXenes have rapidly developed into an important class of materials consisting of two-dimensional transition-metal carbides, nitrides and carbonitrides [12]. Recent reviews have further highlighted that MXenes possess high electrical conductivity, hydrophilic surfaces and tunable layered structures. These features make them suitable not only as electron-transport frameworks, but also as platforms that can regulate the reaction environment through surface terminations, defects, interlayer space and heterointerfaces. For instance, Huang et al. anchored a high-entropy alloy active phase on an MXene support and achieved an overpotential of 267 mV at 10 mA cm^−2^ in alkaline media [13]. Yang et al. reported a Mo_2_CT_x_/NiFe alloy/layered double hydroxides (LDH) heterostructure catalyst, which exhibited an overpotential of 230 mV at 10 mA cm^−2^ in 1.0 M KOH [14]. However, as intensive research on MXenes has progressed, some inherent limitations of MXenes have also been identified, including their limited number of intrinsic active sites, the tendency of nanosheets to restack and oxidative instability under OER conditions [15,16,17]. These shortcomings highlight the necessity for targeted modification of their composition, surface chemistry and hierarchical structure. Through modification strategies such as coupling with active phases, regulating interfacial charge transfer, optimizing mass-transport pathways, and reshaping the coordination and electronic environment of active centers at the atomic level, it becomes possible to enhance the catalytic activity of MXenes [16,18,19]. Wang et al. prepared a (Ni,Fe)S_2_@Ti_3_C_2_ composite by first delaminating Ti_3_AlC_2_ to obtain few-layer Ti_3_C_2_, then by growing NiFe LDH on the Ti_3_C_2_ substrate through reflux coprecipitation, followed by controllable sulfurization [20]. Nazari and Morsali synthesized a Fe_2.1_Ni_0.2_Co_0.7_-MIL-88A/Ti_3_C_2_T_x_ composite through a one-pot solvothermal in situ growth strategy, in which trimetallic MIL-88A particles were grown directly on conductive Ti_3_C_2_T_x_ nanosheets [21].

Recent reviews focused on the role of MXenes in water electrolysis have examined this topic from different perspectives [18,22]. Some have summarized the progress of MXenes by discussing their development in the field of HER, OER and overall water splitting in parallel. Others have focused on strategies such as surface terminations, defects, doping and the incorporation of secondary components. There are also OER-focused reviews emphasizing that MXenes can serve not only as highly conductive frameworks, but also as precursor platforms and interfacial regulators for active phases [17,18]. However, many existing discussions still mainly follow reaction types or material categories, whereas the correspondence between regulation strategy, working-state evolution and OER pathway remains insufficiently clarified [15,16,17,18,23,24]. Unlike previous reviews that are organized mainly by material categories or discuss modification strategies separately, it constructs a systematic framework from low-dimensional and micro-level control to high-dimensional and macro-level integration, covering all levels from atoms and local structures to interfaces, morphology and composite electrodes. It correlates atomic environments, heterointerfaces, morphologies, and composite architectures with changes in electronic structure, active-site accessibility, mass transport, reaction pathways, working-phase reconstruction, and OER durability. Particular attention is given to distinguishing the functions of MXenes as active phases, conductive supports, precursors or interfacial regulators. It further distinguishes the beneficial surface reconstruction that produces catalytically active hydroxyl oxide species from the destructive oxidative hydrolysis that leads to structural collapse, and provides a certain basis for evaluating whether the anode surface reconstruction improves or reduces the catalyst performance. Through this cross-scale analysis, the review aims to clarify structure–activity–stability relationships, identify limitations in current mechanistic assignments, and provide practical guidance for designing efficient, durable, and experimentally verifiable MXene-based OER electrodes.

## 2. OER Mechanisms: AEM, LOM and OPM

OER is the key kinetic process at the anode of water electrolysis and the core step that determines the overall energy-conversion efficiency. Compared with the cathodic HER, OER involves a four-electron-coupled transfer process, the successive evolution of multiple oxygen-containing intermediates and O–O bond formation. It therefore follows a more complex reaction pathway and exhibits more sluggish kinetics, making it the major origin of the overpotential in overall water splitting. For this reason, elucidating the OER mechanism is fundamental to analyzing differences in catalytic activity, identifying the origin of superior performance and guiding materials design. At present, two kinds of OER pathways are well known: AEM and LOM. In this chapter, we will not only explain AEM and LOM in detail, but also introduce a novel OER mechanism named OPM.

### 2.1. AEM

AEM essentially describes the OER process as a stepwise elementary adsorption–conversion process occurring at the surface-active sites of the catalyst. In alkaline media, this mechanism generally proceeds through the stepwise generation and evolution of key intermediates such as *OH, *O and *OOH. Under alkaline conditions, the AEM proceeds through the successive adsorption and conversion of OH*, O* and OOH* intermediates at the surface-active site (*). The four elementary steps can be expressed as follows:

OH^−^ + * → OH* + e^−^ (1)

OH^−^ + OH* → O* + H_2_O + e^−^ (2)

OH^−^ + O* → OOH* + e^−^ (3)

OH^−^ + OOH* → O_2_ + H_2_O + e^−^ + * (4)

**Figure 1:**
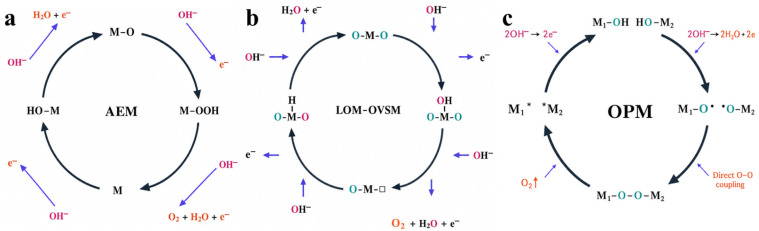
Schematic diagram of (**a**) AEM, (**b**) LOM-OVSM mechanism, and (**c**) OPM of OER. Black curved arrows indicate the progression of the reaction cycles, whereas blue straight arrows indicate reactant addition or product/electron release. Magenta highlights hydroxide-derived oxygen, cyan highlights lattice or oxyl oxygen, and orange highlights released products or electrons. The symbols *, □, and • denote a surface active site, an oxygen vacancy, and an unpaired electron, respectively.

In this pathway, OH^−^ is first adsorbed and oxidized to OH*, followed by deprotonation to form O*. A second OH^−^ then reacts with O* to produce OOH*, after which O_2_ is released and the initial active site is regenerated. The above process is briefly shown in Figure 1a. In this process, catalytic activity is therefore governed by the adsorption free energies of OH*, O* and OOH*. Because AEM directly correlates catalytic activity with the adsorption strength of surface intermediates, it has been the most commonly used theoretical framework in OER mechanistic analysis. However, AEM also has limitations. Excessively strong oxygen binding suppresses OOH* formation or desorption, whereas insufficient oxygen binding increases the energy barrier for OH* deprotonation. Moreover, studies have shown that the adsorption free energies of intermediates such as *OH, *O and *OOH generally follow approximately linear scaling relationships, which means that catalysts usually cannot achieve simultaneous optimization of all elementary steps [6,7]. In other words, even if the adsorption behavior of an intermediate in a given elementary step can be improved by optimizing the surface electronic structure, it is still difficult to overcome the thermodynamic limitation of the overall reaction pathway. Though AEM provides a unified analytical framework for OER, it also reveals the intrinsic bottleneck of the conventional adsorbate pathway in terms of theoretical activity enhancement.

### 2.2. LOM

The LOM extends the reaction center from surface-adsorbed intermediates to the lattice oxygen atoms of the catalyst itself. This mechanism holds that under specific electronic structures and local bonding environments, lattice oxygen is not merely a passive component that maintains framework stability, but may be activated and directly participate in the formation of an O–O bond. Compared with AEM, which mainly relies on the successive evolution of surface-adsorbed intermediates, LOM places greater emphasis on the key roles of the regulation of oxygen electronic states and lattice oxygen activation. Therefore, it is often used to explain the unusual catalytic behavior of some highly active oxide or oxyhydroxide species that deviates from the AEM pathway. For MXene-containing catalysts, this point is particularly relevant because surface terminations, interfacial bonding and reconstructed oxyhydroxide species can jointly affect whether oxygen evolution follows an adsorbate-dominated pathway or involves lattice-oxygen-related participation [6,25,26,27].

LOM involves the direct participation of surface lattice oxygen in O–O bond formation. Taking the representative oxygen-vacancy site mechanism in LOM (LOM-OVSM) shown in Figure 1b as an example, OH^−^ first attacks an activated lattice-oxygen site within the M–O_L_–M configuration, generating an O_L_–OH intermediate. Subsequently, lattice oxygen is coupled with oxygen in the hydroxide radical, forming “O−O”. Then oxygen is released, producing an oxygen vacancy (V_O_). The vacancy is then refilled by OH^−^, followed by deprotonation and reconstruction of the original metal–oxygen lattice. The whole reaction is accompanied by the loss of four electrons. Therefore, unlike AEM, in which O_2_ originates exclusively from adsorbed oxygen intermediates, LOM is accompanied by lattice-oxygen consumption, oxygen-vacancy formation and dynamic surface reconstruction.

Different from AEM, LOM is usually inferred from the pH-dependent activity trend and Tafel slope. LOM involves the direct participation of bulk oxygen or surface lattice oxygen in the formation of O-O bonds. Therefore, conclusive evidence of LOM should ideally include at least two supplementary diagnoses: (i) isotope labeling experiment (for example, using catalyst rich in ^18^O and ^18^O or H_2_^18^O electrolyte) combined with differential electrochemical mass spectrometry (DEMS) to track the isotopic composition of precipitated O_2_; (ii) pH-dependent kinetics, which deviates from the classical first-order dependence on OH activity, and usually shows a reaction order greater than 1 or a non-Nernst pH dependence; (iii) operating spectral signals, such as peroxide-like (O–O) vibration mode, covalent change in the metal–oxygen bond or reduced metal state indicating the formation of oxygen vacancies; and (iv) quantitatively analyzing the released oxygen to distinguish surface oxygen from lattice oxygen.

For MXene-based electrocatalysts, AEM is more likely to predominate when the active oxide or oxyhydroxide phase maintains a stable M–O lattice and contains relatively few oxygen vacancies. LOM becomes more competitive when interfacial charge transfer, surface defects or anodic reconstruction strengthens M–O covalency and activates lattice oxygen. Although LOM can bypass the conventional OOH* scaling constraint and accelerate OER kinetics, repeated lattice-oxygen removal may also cause metal dissolution and structural degradation. Thus, the dominant pathway depends on the balance between adsorbate optimization, lattice-oxygen activity and structural stability [25,26,27].

### 2.3. OPM

Besides AEM and LOM, the OPM has been proposed as a third possible pathway for O–O bond formation. Early studies of anionic oxygen redox demonstrated that O–O bond formation is not necessarily restricted to the conventional metal-centered *OOH pathway. When strong metal–oxygen covalency brings the O 2p states close to the Fermi level, oxygen-centered oxidation and direct coupling between oxidized oxygen species may become accessible [28,29]. In the narrower, vacancy-free definition adopted here, OPM proceeds through the direct coupling of two adjacent metal-bound oxyl species without consuming lattice oxygen.

Under alkaline conditions, two neighboring metal sites, denoted as M_1_* and M_2_*, are first hydroxylated and subsequently deprotonated to generate two surface oxyl radicals. The two neighboring oxyl radicals then undergo direct radical coupling to form a bridging peroxide- or superoxide-like intermediate, followed by O_2_ release and regeneration of the initial sites (Figure 1c). The steps can be expressed as follows:

M_1_* + OH^−^ → M_1_–OH + e^−^ (5)

M_2_* + OH^−^ → M_2_–OH + e^−^ (6)

M_1_–OH + OH^−^ → M_1_–O• + H_2_O + e^−^ (7)

M_2_–OH + OH^−^ → M_2_–O• + H_2_O + e^−^ (8)

M_1_–O• + •O–M_2_ → M_1_–O–O–M_2_ (9)

M_1_–O–O–M_2_ → O_2_ + M_1_* + M_2_* (10)

Here, * denotes an available surface metal site, whereas • denotes an unpaired electron localized on a surface oxyl species.

Unlike AEM, OPM bypasses the formation of the *OOH intermediate and can therefore circumvent the conventional scaling relationship between *OH and *OOH. Unlike vacancy-mediated LOM, the vacancy-free OPM does not require the extraction of lattice oxygen or the formation of oxygen vacancies. Instead, its feasibility is governed by the distance and geometric arrangement between adjacent metal sites, the surface coverage of *OH/*O species, and the electronic coupling between the two active centers. Thus, sufficiently short metal–metal distances, high oxyl coverage, and suitable dual-site coordination are generally favorable for direct O–O radical coupling. Experimental evidence for OPM has recently been obtained for cobalt-based oxides. Wang et al. demonstrated that Ba incorporation shortened the Co–Co distance and increased surface OH coverage in Co_3−x_Ba_x_O_4_. In situ FTIR detected an O–O vibration at 1122 cm^−1^ and a linearly bonded M–O–O species at 1136 cm^−1^, while isotope-labeled operando differential electrochemical mass spectrometry further supported the coupling of oxygen species adsorbed on neighboring Co sites. These results, together with density functional theory (DFT) calculations, indicated that Ba incorporation promoted OPM over the conventional AEM [30].

A related dual-site pathway, termed the diatomic oxygen mechanism (DOM), was reported for Ru-substituted Co_3_O_4_ and can be regarded as a heterometallic realization of OPM. The highly symmetrical and shortened Ru_oct_–O–Co_oct_ coordination enabled direct coupling between oxygen radicals adsorbed on neighboring Ru and Co sites. In situ Raman spectroscopy detected a dioxygen-radical-coupling band at approximately 1050 cm^−1^, whereas the characteristic *OOH signals expected for AEM were absent. The calculated potential-determining-step barrier for this pathway was 1.74 eV, lower than those calculated for AEM and LOM on the same catalyst, namely 1.88 and 2.07 eV, respectively [31]. For MXene-based electrocatalysts, OPM may become competitive at closely coupled dual-metal sites or reconstructed oxyhydroxide/MXene interfaces, where interfacial charge redistribution can regulate metal–metal separation and increase the coverage of reactive *OH and *O species. Nevertheless, OPM should not be assigned solely from a reduced overpotential, an altered d-band center, or a favorable theoretical energy diagram. Convincing identification requires complementary evidence, such as operando detection of O–O or M–O–O intermediates, isotope-resolved oxygen analysis, dependence on active-site separation or surface coverage, and the simultaneous exclusion of *OOH-dominated AEM and vacancy-forming LOM pathways.

In conclusion, AEM and LOM constitute the basic framework for understanding OER, respectively emphasizing the gradual transformation of surface adsorption intermediates and the direct participation of lattice oxygen in the formation of O-O bonds. On this basis, OPM, proposed in recent years as an important supplement, emphasizes that surface metals and adjacent oxygen sites cooperate to form mixed oxygen coupling transition States, which is suitable for highly covalent and easily reconfigurable systems and can explain phenomena such as pH dependence and isotope labeling that AEM/LOM cannot cover. For the MXene system in this review, the performance control strategy should not only affect the behavior of AEM intermediates by adjusting the surface electronic structure and adsorption environment, but also intervene in the LOM trend by changing the metal–oxygen bonding state and oxygen activation ability. At the same time, we should pay attention to the effects of surface terminal, vacancy and strain on oxygen species migration and coupling configuration, so as to comprehensively evaluate the contribution of the OPM path. The integration of AEM, LOM and OPM provides a more complete and distinct mechanism perspective for the subsequent analysis of the reaction path selectivity, activity source and stability of MXene-based catalysts.

## 3. Modification Strategy of MXene-Based Catalyst

Although MXene-based OER catalysts are modified through different routes, their performance enhancement generally originates from several common principles. Regulation of the local electronic structure changes charge density, orbital occupation, and metal–oxygen bonding, thereby adjusting the adsorption energies of *OH, *O, and *OOH. Increasing the specific surface area and suppressing MXene restacking expose more accessible sites, while suitable porosity, wettability, and interconnected transport channels facilitate OH^−^ supply and O_2_ release. Meanwhile, the number and intrinsic strength of active sites should be optimized simultaneously, because additional surface area is not necessarily beneficial when the newly exposed surface is inactive or blocks the catalytic phase. In addition, the catalyst can be optimized by reducing the interfacial resistance, balancing the adsorption of reactants and the desorption of products, reducing the energy demand of the potential determination step, optimizing the reaction path, promoting the formation of the actual working phase, and strengthening the structural stability of the catalyst. These effects are usually coupled rather than independent. For example, Ma et al. showed that Ru/V codoping and MXene coupling shifted the d-band center and optimized intermediate adsorption [32]. Aboelazm et al. found that Co 3d–Se 4p–Ti 3d coupling increased the electronic states near the Fermi level and promoted interfacial charge transfer [33]. Accordingly, effective catalyst design should integrate multiple complementary principles rather than optimize a single parameter in isolation. Excellent designs should employ various design principles as much as possible.

Different from reviews that are mainly organized according to material category or a single strategy, this review relates the degree of material modification, the changes in physical and chemical parameters and the resulting OER behavior. Therefore, it constructs a framework from low-dimensional and micro-level control to high-dimensional and macro-level integration, covering all levels from atoms and local structures to interfaces, morphology and composite electrodes. This chapter is divided into four strategy-based subsections, each emphasizing a different regulatory range and a different combination of the common principles described above. Section 3.1 focuses on the lowest-dimensional atomic regulation. Dopants, vacancies, surface terminations, and defined atomic sites directly modify local coordination, orbital occupation, adsorption energies, and reaction pathways. Section 3.2 examines interface engineering, which acts at phase boundaries. Interfacial bonding and charge redistribution improve electronic transport, alter intermediate adsorption, accelerate formation of working active species, and stabilize catalytic sites. Section 3.3 addresses morphology engineering at the particle and electrode-architecture scale, mainly increasing accessible area, controlling active-site exposure, shortening diffusion distances, and facilitating electrolyte infiltration and O_2_ release. Section 3.4 considers composite engineering as the broadest systems-level method. It integrates active phases, conductive networks, stabilizing components, and self-supporting substrates to improve charge transport, mass transfer, accessible area, and durability simultaneously. Taken together, the following sections establish a comparative dimensional map in which atomic environments govern intrinsic activity, interfaces control interphase electronic communication, morphology determines accessibility and transport, and composite architectures coordinate these effects at the electrode level.

### 3.1. Vacancy and Other Atomic-Level Regulation

This section focuses on the core issue at the level of local environment regulation, namely how the coordination structure, valence-state distribution and electron density in the vicinity of active sites can be precisely optimized through doping, vacancy engineering and the construction of atomic-level sites. MXenes possess abundant surface terminations, tunable local coordination environments and strong electronic-structure tunability. Therefore, foreign-element incorporation, vacancy enrichment and the anchoring of single-atom and dual-atom sites can all construct new local reaction microenvironments around the active sites. Local environment regulation mainly affects OER kinetics and catalytic activity by altering OH^−^ adsorption, the conversion of oxygen-containing intermediates and interfacial charge-transfer processes.

Chen et al. reported that NH_3_/Ar plasma treatment simultaneously induced flake delamination, active-site generation and nitrogen incorporation in Ti_3_C_2_T_x_, ultimately yielding few-layer Ti_3_C_2_T_x_-N_y_ with tunable nitrogen doping. The results showed that when the NH_3_:Ar volume ratio reached 6:1, Ti_3_C_2_T_x_-N_6_ exhibited the largest c lattice parameter of 13.53 Å, the highest nitrogen content of 1.57 wt% and the highest proportion of surface-adsorbed N (SA), reaching 64.34%. In electrochemical testing, Ti_3_C_2_T_x_-N_6_ in 1.0 M KOH required potentials of 1.59 and 1.74 V to reach current densities of 10 and 100 mA cm^−2^, corresponding to overpotentials of 360 and 510 mV, respectively. Its Tafel slope was 76.68 mV dec^−1^ and its double-layer capacitor (C_dl_) reached 1.58 mF cm^−2^. Notably, these results indicate that nitrogen doping not only altered the elemental composition, but was also accompanied by regulation of the local chemical environment on the Ti_3_C_2_T_x_ surface. The mechanistic analysis further indicated that the plasma process promoted delamination through the instantaneous release of interlayer gases generated by NH_4_HCO_3_ decomposition, while simultaneously creating more surface active sites and enabling nitrogen incorporation through the combined effects of Ar-ion bombardment and nitrogen-containing reactive species. Overall, nitrogen incorporation changed the local electron distribution, delamination state, active-site population and charge-transfer behavior of the material and these changes corresponded to its improved OER response [34].

**Figure 2:**
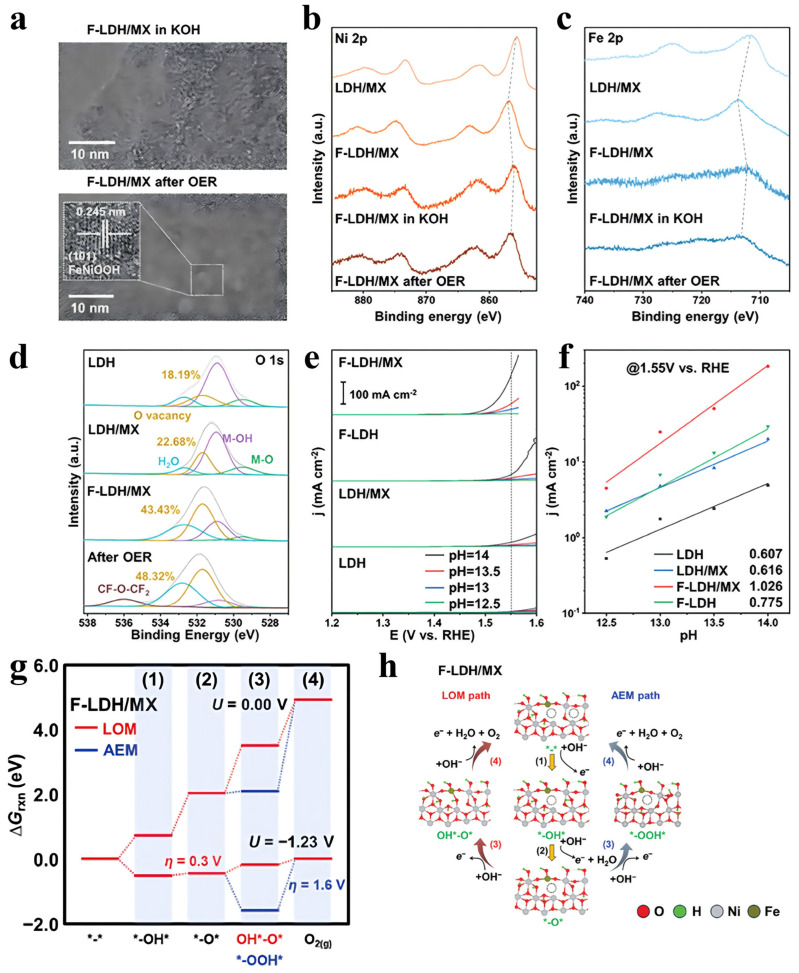
(**a**) The transmission electron microscope (TEM) images of F-LDH/MX in KOH and F-LDH/MX after OER. In (**a**), the outlined region and connecting lines indicate the area displayed in the HRTEM inset. The high-resolution spectra of (**b**) Ni 2p and (**c**) Fe 2p for LDH/MX, F-LDH/MX, F-LDH/MX in KOH, and F-LDH/MX after OER. The dashed guide lines in (**b**,**c**) mark the corresponding Ni 2p and Fe 2p peak positions. (**d**) The high-resolution spectra of O 1s for LDH, LDH/MX, F-LDH/MX, and F-LDH/MX after OER. (**e**) OER polarization curves of LDH, LDH/MX, F-LDH, and F-LDH/MX in KOH electrolyte at 5 mV s^−1^ from pH 12.5 to 14 on the RHE scale. (**f**) OER specific activities of all catalysts at 1.55 V versus different pHs of KOH. In (**d**–**f**), the colored curves correspond to the fitted components or catalyst samples identified by the respective in-panel labels and legends. (**g**) DFT-calculated reaction energies of the OER at an applied potential of U = 0.00 V and U = −1.23 V for the activated F-LDH/MX. (**h**) The LOM and AEM pathways are calculated for activated F-LDH/MX. The overpotential(η) of each reaction pathway is denoted for the corresponding potential-determining step. The color coding for the elements is as follows: O (red), H (green), Ni (gray), and Fe (olive). In (**g**,**h**), the red and blue pathways represent LOM and AEM, respectively, and (1)–(4) label the successive reaction steps. The symbol * denotes a surface adsorption site; an asterisk attached to a chemical species indicates an adsorbed intermediate, whereas paired asterisks denote two neighboring surface sites. Reprinted from Ref. [27].

Atomic-level engineering represents the lowest-dimensional regulation because it directly changes surface bonds, local coordination, vacancy concentration, and the electronic state of individual catalytic centers. Pei et al. prepared LDH/MX through the coprecipitation of Fe and Ni species in the presence of few-layer Ti_3_C_2_T_x_ and subsequently fluorinated the catalyst surface under controlled thermal conditions [27]. After immersion in 1.0 M KOH without an applied potential, F-LDH/MX becomes largely amorphous; after OER, a lattice spacing of 0.245 nm assigned to NiFeOOH appears together with abundant holey defects, directly showing conversion of the fluorinated precursor into a reconstructed oxyhydroxide phase (Figure 2a). The Ni 2p_3/2_ peak shifts from approximately 856.76 to 856.08 eV upon KOH immersion, consistent with spontaneous defluorination and Ni(OH)_2_ formation; after OER, it shifts to higher binding energy, supporting further oxidation to Ni oxyhydroxide (Figure 2b). The Fe 2p spectra show the same sequence, namely Fe hydroxide formation in KOH followed by Fe oxyhydroxide formation under OER polarization (Figure 2c). O 1s deconvolution quantifies the oxygen-vacancy fraction as 18.19% for LDH, 22.68% for LDH/MX, 43.43% for F-LDH/MX, and 48.32% after OER (Figure 2d). Electrochemically, F-LDH/MX requires 251 mV at 10 mA cm^−2^ and exhibits a Tafel slope of 40.28 mV dec^−1^; its polarization curves before and after 1000 cycles nearly overlap, and no significant current loss is observed after 30 h. The pH-dependent polarization curves show the strongest current response to pH for F-LDH/MX (Figure 2e), and the corresponding slope, ρ = (∂logi/∂pH)E, reaches 1.026, compared with 0.607 for LDH, 0.616 for LDH/MX, and 0.775 for F-LDH (Figure 2f). DFT calculations give overpotentials of 0.3 V for LOM and 1.6 V for AEM on activated F-LDH/MX (Figure 2g), and the corresponding pathways are compared at the reconstructed surface (Figure 2h). Taken together, the reconstructed NiFeOOH lattice, increased vacancy fraction, strong pH dependence, and lower calculated LOM overpotential support enhanced lattice-oxygen participation. However, because isotope-labeled oxygen analysis was not performed, these results do not prove an exclusive LOM pathway. The reported O_2_ Faradaic efficiency of 93.71% confirms that most anodic charge is used for oxygen evolution. Therefore, MXene mainly provides a conductive and electronic-regulation platform, whereas the fluorinated and reconstructed NiFe oxyhydroxide surface supplies the working OER-active sites.

Ma et al. reported that coupling V-doped Co_2_P with high-entropy MXene produced the V-Co_2_P@HE heterostructure. XAS and XPS analyses jointly indicated that V was incorporated into the Co_2_P lattice in the form of dopant species and participated in tuning the local electronic environment. Specifically, the high-resolution Co 2p XPS spectra showed that the Co peaks in V-Co_2_P@HE shifted by about 0.3 eV toward higher binding energy relative to those of Co_2_P, indicating that V doping and heterointerfacial coupling jointly altered the electronic environment around Co. Further Co K-edge XANES analysis showed that the average valence state of Co in V-Co_2_P@HE lay between 0 and +2 and was closer to +2. Together with the Co K-edge extended X-ray absorption fine structure (EXAFS) and wavelet-transform results, this suggested that the local coordination structure around Co had been reconfigured. Meanwhile, the V K-edge XANES and EXAFS results further indicated that V mainly entered the Co_2_P lattice in a non-zero-valence state rather than existing as an independent metallic phase, single atoms or clusters. Part of the surface-exposed V may be oxidized to VO_x_ and continue to influence the local electronic and coordination environments. In 1 M KOH, V-Co_2_P@HE required an overpotential of only 227 mV to reach 10 mA cm^−2^ and exhibited a Tafel slope of 44.45 mV dec^−1^. When the current density was increased to 150 mA cm^−2^, its overpotential remained as low as 327 mV. The collected H_2_/O_2_ volume ratio was close to 2:1 and the Faradaic efficiency reached 94.8%, confirming that water splitting was the predominant reaction. Post-OER XPS showed that the metal–P contribution almost disappeared and new metal–O bonds emerged, while TEM revealed an amorphous cobalt-oxide layer on the catalyst surface. These results demonstrate that V-Co_2_P@HE functions as a reconstructing precatalyst and that the derived oxide phase constitutes the working OER surface. Subsequently, V-Co_2_P@HE was continuously tested at 100 mA cm^−2^ for 48 h. Researchers further used DFT calculations to compare the H_2_O adsorption energies of Co_2_P, V-Co_2_P and V-Co_2_P@HE and analyzed the free-energy changes along the OER pathway on the basis of reconstructed surface models. Combined with the density of states (DOS), d-band center and differential charge-density analyses, the results confirmed that V doping and electronic coupling at the heterogeneous interface jointly enhanced the adsorption and activation of water molecules and lowered the reaction barrier of the reconstructed OER process. Taken together, the experimental characterizations and theoretical calculations show that after V entered the lattice, the electron density, valence-state distribution and local coordination environment around Co were all re-regulated, which further optimized the adsorption behavior of OER intermediates at the Co active sites [35]. This example also shows that dopant regulation in MXene-based OER systems can operate together with interfacial electronic coupling, rather than serving as an isolated compositional modification [36,37,38,39,40].

Li et al. found that the surface hydroxyl groups of monolayer MXene could attract Ce ions and induce the formation of surface defects during Ce-MOF growth, thereby further promoting the generation of oxygen vacancies. The initial XPS results showed that a new O 1s component assigned to oxygen vacancies appeared in MXene@Ce-MOF. After galvanostatic charge–discharge cycling, the proportion of oxygen vacancies increased further. Meanwhile, after charge–discharge cycling, the peak of surface chemisorbed oxygen disappeared and a new lattice-oxygen feature emerged at 529.1 eV. In the Ce 3d spectrum, a new Ce^4+^ characteristic peak also appeared at 898.1 eV, indicating that the generation of higher-valent Ce species occurred synchronously with lattice-oxygen activation. In 1.0 M KOH, MXene@Ce-MOF required an OER overpotential of 270 mV to reach 10 mA cm^−2^, which was markedly lower than those of Ce-MOF (348 mV) and MXene (357 mV). The Tafel slope of MXene@Ce-MOF was 163.8 mV dec^−1^. The OER activity retention remained as high as 99.7% after 12 h of stability testing. The study further pointed out that oxygen vacancies could donate electrons to the system, lower the Fermi level during the reaction, improve the surface hydrophilicity of the catalyst and facilitate electron transport and exchange between active sites [41]. Other oxygen-vacancy-rich MXene heterostructures further support the conclusion that vacancy engineering usually acts together with interfacial charge redistribution and morphological exposure, rather than functioning as an isolated defect effect [42,43,44,45].

Chen et al. adopted Ar/H_2_ plasma treatment to convert Co_9_S_8_/Ti_3_C_2_T_x_ into Co_9_S_8-x_/Ti_3_C_2_T_x_. The introduction of sulfur vacancies was accompanied by changes in the local coordination environment and electronic structure. Raman results showed that the D/G intensity ratio increased from 0.86 to 0.94, indicating a higher defect density. High-resolution XPS of Co 2p showed that the Co^2+^/Co^3+^ ratio decreased from 0.94 to 0.90, suggesting a further shift in the local electronic environment. Electron paramagnetic resonance (EPR) displayed a characteristic signal at g = 2.005, directly confirming the presence of sulfur vacancies; and BET analysis showed that the specific surface area increased from 89.7 to 159.8 m^2^ g^−1^. Morphological characterization further showed that Ti_3_C_2_T_x_ incorporation suppressed the severe agglomeration of Co_9_S_8_, while the subsequent Ar/H_2_ plasma treatment rendered the composite more porous and reduced the Co_9_S_8_ particle size to about 20–30 nm, as confirmed by the scanning electron microscope (SEM) and TEM images. In terms of catalytic performance, the overpotential at 10 mA cm^−2^ decreased from 381 mV for pure Co_9_S_8_ to 342 mV for Co_9_S_8_/Ti_3_C_2_T_x_ and further to 286 mV for Co_9_S_8-x_/Ti_3_C_2_T_x_. The Tafel slope of Co_9_S_8−x_/Ti_3_C_2_T_x_ was 76 mV dec^−1^. Furthermore, its R_ct_ value was only 7.1 Ω and the C_dl_ value reached 55.2 mF cm^−2^. The OER Faradaic efficiency determined by the water-displacement method was 90.06%, confirming that oxygen evolution was the predominant anodic reaction, although a minor contribution from catalyst oxidation or other processes could not be excluded. Post-OER HRTEM and XRD further revealed the formation of CoOOH from the Co_9_S_8−x_ surface, demonstrating that the sulfur-deficient sulfide served as a reconstructing precatalyst. In this precatalyst, Ti_3_C_2_T_x_ should act primarily as a conductive and dispersing carrier, while CoOOH formed through the restructuring of Co_9_S_8−x_ during the OER process serves as the main phase responsible for OER activity. Under continuous operation at 100 mA cm^−2^ for 50 h, the overpotential rose only from 333 to 349 mV. Subsequent high-resolution transmission electron microscope (HRTEM) analysis also revealed two sets of lattice fringes with spacings of 0.30 and 0.15 nm, corresponding to the (311) plane of Co_9_S_8_ and the (110) plane of CoOOH, respectively, indicating that CoOOH surface species formed in the vacancy-enriched system during prolonged OER operation [46].

Khoshfetrat et al. reported that Ti_3_C_2_ was first converted into a Ti_3_C_2_/TiO_2_ framework through controlled oxidation, after which CoMoO_4_ was grown in situ and subsequently subjected to heat treatment under N_2_ to yield p-Ti_3_C_2_/TiO_2_–CoMoO_4_, thereby extending oxygen-vacancy engineering to the level of composite local environment regulation. The study provided a clear structural-evolution pathway: the (002) plane of Ti_3_C_2_ corresponded to an interlayer spacing of about 0.96 nm, which expanded to 1.67 nm after controlled oxidation and further increased to 3.53 nm after CoMoO_4_ incorporation. In the BET measurements, the specific surface area rose from 8.2 m^2^ g^−1^ for Ti_3_C_2_ to about 28, 76.7 and 107.6 m^2^ g^−1^ for Ti_3_C_2_/TiO_2_, Ti_3_C_2_/TiO_2_–CoMoO_4_ and p-Ti_3_C_2_/TiO_2_–CoMoO_4_, respectively. More crucial evidence came from EPR: all samples exhibited an oxygen-vacancy signal at g ≈ 2.003 and the signal intensity followed the order CoMoO_4_ < Ti_3_C_2_ < Ti_3_C_2_/TiO_2_ < Ti_3_C_2_/TiO_2_–CoMoO_4_ < p-Ti_3_C_2_/TiO_2_–CoMoO_4_, indicating that the heat-treated composite possessed the highest oxygen-vacancy concentration. In 1 M KOH, p-Ti_3_C_2_/TiO_2_–CoMoO_4_–NiNC–NF delivered an overpotential of 190 mV at 10 mA cm^−2^ with a Tafel slope of about 56.1 mV dec^−1^ and an exchange current density of 96.25 mA cm^−2^. Moreover, its C_dl_ was as high as 408.2 mF cm^−2^. During a 200 h chronopotentiometric testing, the potential increased by only 2.82% at 10 mA cm^−2^ and 3.31% at 100 mA cm^−2^. However, the authors pointed out that although the OER enhancement of the optimized catalyst was partly associated with its larger electrochemically active surface area, the dominant contribution arose from the intrinsic-activity gain induced by heterogeneous interface electron coordination and the vacancy-rich local environment, with about 43.6% of the improvement originating from surface-area expansion and about 56.4% from interfacial electronic synergy. Oxygen vacancies, partially reduced Co/Mo sites and heterointerfacial electronic synergy jointly promoted local charge delocalization and regulated OH^−^ adsorption, thereby translating local defect states into lower reaction barriers and stronger adaptability under high-current conditions [47].

**Figure 3:**
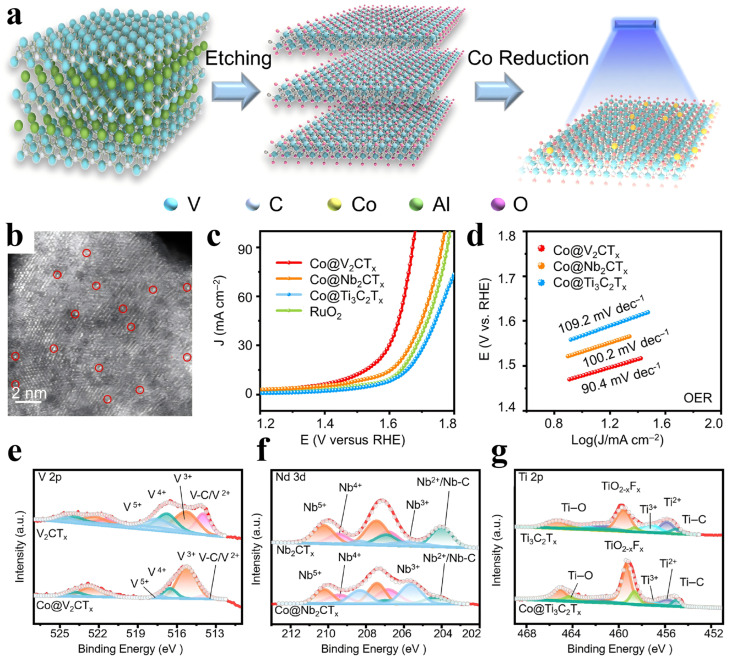
(**a**) Schematic illustration of the fabrication of Co@MXene composites on an example of V_2_CT_x_; (**b**) HAADF-STEM image of the surface of Co@V_2_CT_x_, with bright spots corresponding to single-atom Co marked by red circles; (**c**) OER LSV curves of Co@V_2_CT_x_, Co@Nb_2_CT_x_, Co@Ti_3_C_2_T_x_ and RuO_2_ electrodes; (**d**) OER Tafel slopes of Co@V_2_CT_x_, Co@Nb_2_CT_x_ and Co@Ti_3_C_2_T_x_ electrodes; (**e**) high-resolution V 2p XPS spectra of Co@V_2_CT_x_ and V_2_CT_x_; (**f**) high-resolution Nb 3d XPS spectra of Co@Nb_2_CT_x_ and Nb_2_CT_x_; (**g**) high-resolution Ti 2p XPS spectra of Co@Ti_3_C_2_T_x_ and Ti_3_C_2_T_x_. Reprinted from Ref. [48].

Atomic-level site engineering was further extended to a finer scale. In the work of Zhao et al., V_2_AlC, Nb_2_AlC and Ti_3_AlC_2_ were used as precursors (Figure 3a). The three MXene materials, namely V_2_CT_x_, Nb_2_CT_x_ and Ti_3_C_2_T_x_, were prepared through LiF-HCl etching (Figure 3a). Researchers then used an iced photochemical reduction method to anchor Co single atoms onto the MXene surface: an aqueous CoCl_2_ solution with a concentration of 0.3 mg mL^−1^ was first frozen into ice cubes, which were then added to a 0.5 mg mL^−1^ MXene dispersion and kept at 0 °C for 1 h, allowing Co^2+^ ions to be released continuously at a low concentration during the slow melting process, thereby suppressing the nucleation of Co metal clusters (Figure 3a). After another 1 h of slow melting at 0 °C, the remaining ice cubes were removed and the mixture was irradiated with UV light at 254 nm and 10 W for 1 h, so that Co^2+^ was reduced and uniformly deposited as single atoms on the MXene substrates, finally yielding the three single-atom catalysts Co@V_2_CT_x_, Co@Nb_2_CT_x_ and Co@Ti_3_C_2_T_x_ (Figure 3a). High-angle annular dark-field imaging scanning electron microscope (HAADF-STEM), XRD and energy dispersive X-ray spectroscopy (EDX) results further demonstrated that Co was distributed over the MXene surfaces in the form of highly dispersed single atoms (Figure 3b). Co@V_2_CT_x_ exhibited an OER overpotential of 242 mV at 10 mA cm^−2^ in 1.0 M KOH, with an R_ct_ of 37.3 Ω and a C_dl_ of 113.5 mF cm^−2^ (Figure 3c). The OER Tafel plots further showed that the reaction kinetics also varied with the MXene substrate, consistent with the substrate-dependent differences in overpotential among the three Co@MXene catalysts (Figure 3d). In addition, high-resolution XPS results showed that the main V 2p peak in Co@V_2_CT_x_ shifted slightly toward higher binding energy relative to that in V_2_CT_x_, whereas the Nb 3d and Ti 2p spectra of Co@Nb_2_CT_x_ and Co@Ti_3_C_2_T_x_ revealed a more pronounced increase in the average valence states of Nb and Ti (Figure 3e–g). On this basis, researchers proposed that V became partially oxidized after the anchoring of Co single atoms, which may originate from electron transfer from V_2_CT_x_ to surface-terminated O. These electronic-state changes further indicate that Co single atoms can redistribute the local charge density at the interface between the Co atom and the MXene substrate. This behavior was attributed to the stronger hybridization between Co 3d and surface-terminated O 2p orbitals, together with more pronounced interfacial charge transfer, which optimized the electronic structure of the Co single atoms and rendered the adsorption and desorption of reaction intermediates more favorable. Further Co K-edge Morlet wavelet transform (MWT) results showed that the Co sites in Co@V_2_CT_x_, Co@Nb_2_CT_x_ and Co@Ti_3_C_2_T_x_ all exhibited two maxima, corresponding to O and neighboring transition-metal atoms in the first and second coordination shells around Co, respectively. Moreover, their MWT patterns were clearly different from those of Co foil, Co_3_O_4_ and CoO, confirming that different MXene substrates indeed altered the local environmental structure of the Co single atoms. In this study, the OER active site was located at the local single-atom site. By varying the MXene substrate, researchers revealed the relationship between the local coordination environment of the single atom and the catalytic performance, showing that when the coordination and electronic environment around the single atom were adjusted, indicators such as overpotential, R_ct_ and C_dl_ also changed accordingly [48].

Zhao et al. further anchored Co/Ni dual-atom sites on the surface of L-tryptophan-premodified Ti_3_C_2_T_x_, thereby obtaining CoNi-Ti_3_C_2_T_x_. HAADF-STEM images displayed well-dispersed bright spots and some Co−Ni dual-atom pairs showed an average interatomic distance of about 0.37 nm. Inductively coupled plasma emission spectrometer (ICP-OES) indicated a metal loading of about 5.6 wt% for CoNi-Ti_3_C_2_T_x_, which is markedly higher than that commonly reported for MXene-based atomic-level systems. Morlet wavelet transform analysis and EXAFS further showed that Co and Ni were mainly coordinated with surface-terminated O and N atoms, while the Ni sites also possessed a more stable second-shell coordination structure. CoNi-Ti_3_C_2_T_x_ required an overpotential of 241 mV to reach 10 mA cm^−2^ and exhibited a Tafel slope of 79.8 mV dec^−1^. After continuous operation for 100 h at 10 and 500 mA cm^−2^, the activity decays were only 1% and 5.1%, respectively. DFT results show that when cobalt and nickel are co-anchored, the degree of electron transfer is higher and cobalt and nickel contribute 0.41 and 0.08 electrons, respectively, indicating that the synergistic interaction between the two sites leads to more obvious charge transfer. This study shows that diatomic sites further extend the local environmental regulation from isolated monometallic centers to adjacent bimetallic units, in which the electronic coupling between adjacent sites and their synergistic regulation of different intermediates jointly affect the multi-step reaction process. The role assignment in these atomically dispersed systems differs from that in reconstructable LDH, sulfide, or phosphide catalysts. The anchored Co and Co–Ni centers constitute the proposed local OER sites, whereas the MXene substrate stabilizes the atoms and regulates their coordination and electronic structures through metal–termination interactions. These results do not establish the entire MXene basal plane as intrinsically active; rather, they show that MXene forms part of a defined active-site environment by coordinating and electronically modulating the dispersed metal centers [49].

These atomic-site studies further indicate that the identification of active sites in MXene-based OER catalysts should consider both the initially anchored atoms and the possible evolution of their local coordination environment under OER conditions [50,51,52]. Fu et al. carried out a theoretical screening of MXene-based single-atom OER catalytic systems through high-throughput first-principles calculations and machine learning and proposed the corresponding structure–activity design principles. In this study, vacancies in the transition-metal layer of MXenes were regarded as single-atom sites and the results showed that a moderate electron-deficient state and a high degree of metal–carbon bond covalency were the key features for achieving high OER activity; machine learning analysis further identified the Bader charge and the M-C bond order as the core descriptors closely correlated with the theoretical overpotential. Local environment regulation mainly centers on changes in coordination structure, electron distribution and adsorption behavior [53].

Beyond isolated dopants and defects, atomic-level engineering can also be achieved by regulating the spatial ordering of different transition metals within the MXene lattice. Here, ordering refers to the chemical distribution of metal atoms between the outer and middle metal layers of an individual MXene slab, rather than the macroscopic alignment or stacking of separate nanosheets. Tan et al. systematically investigated eight ternary M_3_C_2_ MXene alloys using high-throughput density functional theory, cluster expansion, and Monte Carlo simulations. Mo-containing alloys exhibited a strong interlayer-ordering tendency, with Mo preferentially occupying the outer metal layers, whereas Nb and Ta tended to occupy the middle layer when alloyed with Ti. In contrast, Ti–V alloys were more likely to form disordered solid solutions. The degree of ordering decreased with increasing temperature, while postsynthesis annealing at 800–1000 K was predicted to restore nearly complete interlayer ordering in several systems. Moreover, electronic-structure analysis demonstrated that changing the metal-layer sequence altered the density of states and metal–carbon bonding environment [54]. For OER electrocatalysis, these results indicate that alloy composition alone cannot fully describe the surface electronic environment. Because the outer metal layers directly interact with surface terminations, electrolyte species, and deposited catalytic phases, controlled ordering may selectively position a regulatory metal at the exposed surface while retaining another metal in the middle layer to stabilize the lattice and mediate electron transport. It therefore provides a more spatially defined form of atomic regulation than conventional random doping and may influence metal–oxygen covalency, intermediate adsorption, and surface reconstruction. Nevertheless, the study by Tan et al. did not directly evaluate OER activity. The catalytic contribution of ordering should therefore be confirmed by comparing ordered and disordered MXenes with identical compositions, together with operando identification of their working surface states.

Based on the representative studies discussed above, nonmetal doping mainly affects the surface chemistry and electronic environment of the MXene substrate itself, whereas metal doping further extends this regulation into the lattice of the active phase. Doping and vacancies influence the reaction behavior by altering local coordination units and electronic structure, while single-atom and dual-atom sites advance the regulation scale toward more explicitly defined local reaction centers. To enable a more intuitive comparison of different atomic-level regulation strategies, Table 1 was compiled to compare the OER activity, kinetics, and stability of representative MXene-based catalysts.

### 3.2. Interface Engineering

MXenes combine a two-dimensional layered structure, metallic conductivity and abundant surface terminations. Therefore, they are often introduced into OER catalytic systems as conductive substrates, interfacial regulators, or composite supports. When coupled with LDHs, chalcogenides, oxides, or other active components, they can form a variety of OER catalysts with rich heterostructures. In addition to the representative systems discussed below, related MXene-based OER studies have extended interface engineering to hydroxide-, oxide-, phosphide-, sulfide-, and selenide-coupled systems. These studies indicate that MXenes are used not only to improve conductivity, but also to regulate active-phase dispersion, interfacial charge redistribution and working-state evolution [56,57,58,59,60,61,62,63,64,65,66,67,68]. In interface engineering, the regulatory mechanisms of catalysts mainly center on interfacial charge transfer, modulation of the electronic structure, the balance between the adsorption and conversion of intermediates, the formation of active species under operating conditions, as well as improvements in charge transport and structural stability.

**Figure 4:**
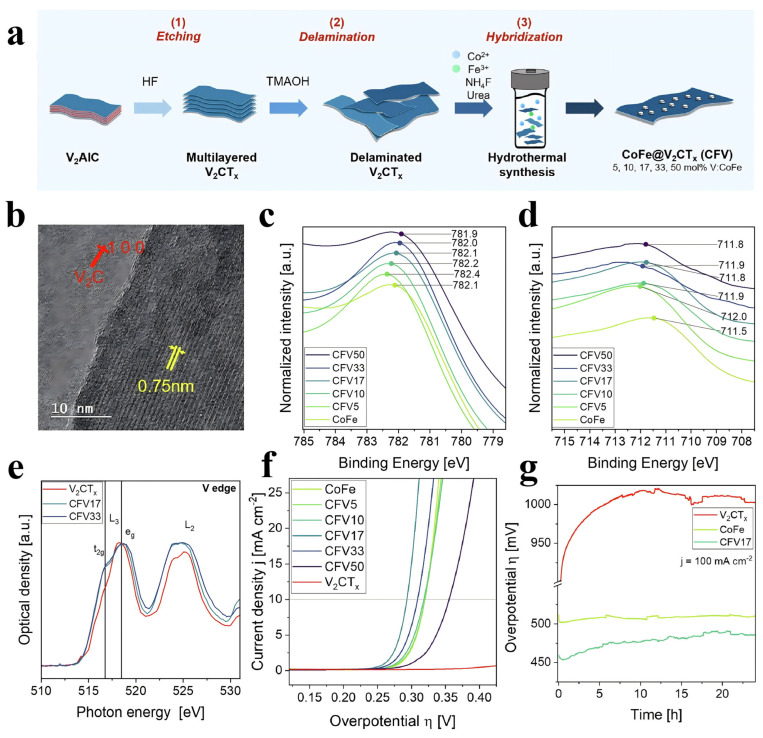
(**a**) Schematic of the three-step synthesis procedure: (1) HF/HCl etching, (2) TMAOH delamination, (3) hydrothermal hybridization. (**b**) HRTEM. (**c**) Co 2p_3/2_. (**d**) Fe 2p_3/2_. (**e**) V L-edge spectra of V_2_CT_x_, CFV17 and CFV33. (**f**) iR-corrected Linear sweep voltammograms of V_2_CT_x_, CoFe, and CFV5~CFV50. The current is normalized by the geometric area of the working electrode. (**g**) Chronopotentiometry of CoFe; CFV17 and V_2_CT_x_ at 100 mA cm^−2^ over 24 h on a 1.0 cm^2^ Ni fiber felt substrate with 1 mg cm^−2^ catalyst loading. Reprinted from Ref. [69].

Kaplan et al. synthesized a series of CoFe@V_2_CT_x_ catalysts with different V_2_CT_x_ contents, denoted as CFV5~CFV50 [69]. Multilayer V_2_CT_x_ was first obtained from V_2_AlC by etching and was subsequently delaminated using TMAOH (Figure 4a). Co and Fe precursors were then introduced during hydrothermal treatment to anchor CoFe species on the delaminated MXene surface. The HRTEM image of the optimized CFV17 sample shows the coexistence of the V_2_CT_x_ framework and a lattice spacing of approximately 0.75 nm assigned to the (001) plane of tetragonal FeOOH (Figure 4b). Because this spacing was absent from pure CoFe, its appearance supports preferential FeOOH nucleation at the V_2_CT_x_ interface. The Co 2p_3/2_ binding energy exhibits a loading-dependent shift relative to CoFe: +0.24 eV for CFV5, +0.11 eV for CFV10, −0.06 eV for CFV17, −0.16 eV for CFV33, and −0.21 eV for CFV50 (Figure 4c). The positive shifts at low V_2_CT_x_ loadings indicate electron withdrawal from Co by the electronegative F, OH and O surface terminations, whereas the reversal from CFV17 onward is consistent with electron donation from the increasingly dominant metallic MXene scaffold. In contrast, the Fe 2p_3/2_ peak shifts from 711.5 eV in CoFe to 712.0 eV in CFV5 and remains positively shifted across CFV5~CFV50, indicating that Fe remains comparatively electron deficient throughout the series (Figure 4d). The V L_3_ peak further shifts from 518.2 eV in V_2_CT_x_ to 518.7 eV in CFV17, while the decreased L_3_/L_2_ branching ratio indicates V oxidation and the increased relative e_g_ contribution, with the t_2g_:e_g_ intensity ratio changing from 1:0.62 for V_2_CT_x_ to 1:0.81 for CFV17 and 1:0.84 for CFV33, suggests enhanced V–O orbital hybridization (Figure 4e). The V L-edge spectra further show changes in the V oxidation state and enhanced V–O orbital hybridization after coupling with CoFe (Figure 4e). Together with preferential FeOOH formation, these observations support V–O–Fe bridging as the most consistent explanation for the interfacial electronic communication. The polarization curves show that CFV17 requires an overpotential of 304 ± 7 mV at 10 mA cm^−2^, compared with 329 ± 9 mV for pure CoFe (Figure 4f). During the 24 h test at 100 mA cm^−2^, CFV17 maintained a lower overpotential than pure CoFe, although a gradual potential increase was still observed (Figure 4g). Therefore, this case demonstrates that an optimum MXene content must balance interfacial conductivity, CoFe-site accessibility, and stabilization of the working FeOOH-containing phase.

Interfacial charge regulation in the heterostructure not only affects electron transport, but also further alters the adsorption behavior of reaction intermediates. Chen et al. used V_2_AlC as the precursor and obtained few-layer V_2_C nanosheets through HF etching, TPAOH intercalation and ultrasonic exfoliation. Subsequently, Ni(NO_3_)_2_·6H_2_O and Fe(NO_3_)_3_·9H_2_O precursors, together with an alkaline solution containing hypophosphite, were simultaneously introduced into the V_2_C dispersion, and NH_4_F was added to regulate the morphology of the LDH crystals. After a hydrothermal process, the H_2_PO_2_^−^/FeNi-LDH-V_2_C heterostructure was obtained. In 1.0 M KOH, this catalyst delivered an overpotential of 250 mV to achieve 10 mA cm^−2^ with a Tafel slope of 46.5 mV dec^−1^. The evolved oxygen was additionally evaluated by rotating ring–disk electrode measurements. The Faradaic efficiency reached 99.2% at 1.48 V and exhibited an average value of approximately 96% between 1.48 and 1.50 V, confirming that most of the measured anodic current originated from oxygen evolution rather than oxidation of the hypophosphite-containing precursor. Electrochemical impedance spectroscopy (EIS) further showed that H_2_PO_2_^−^/FeNi-LDH-V_2_C exhibited the lowest charge-transfer resistance among the prepared catalysts, indicating a faster interfacial charge-transfer process after the introduction of V_2_C. H_2_PO_2_^−^/FeNi-LDH-V_2_C was subjected to an OER stability test at a constant overpotential of 0.25 V, and no obvious decay in current density was observed within 30,000 s. Furthermore, after a 50,000 s OER test, the catalyst was further characterized by XRD, TEM, elemental mapping and XPS. The results showed that the XRD peaks remained largely similar before and after the reaction, while the flaky morphology and elemental distribution were overall preserved, indicating good structural stability of the catalyst. After the reaction, the V 2p spectrum still corresponded to V^4+^ species. In contrast, the Ni 2p spectrum displayed new characteristic peaks assigned to Ni^3+^, indicating that Ni^2+^ was further oxidized to Ni^3+^ during OER. Meanwhile, the earlier electron energy-loss spectroscopy (EELS) results showed that H_2_PO_2_^−^/FeNi-LDH-V_2_C possessed a higher Fe L_3_/L_2_ integral intensity ratio, indicating that its Fe species were in a higher valence state among fresh samples. After the 50,000 s OER test, the Fe 2p spectrum further exhibited a slight positive shift, suggesting that the valence state of the Fe species increased further during the reaction. Interfacial charge was transferred from the LDH phase to V_2_C, accompanied by increased valence states of Ni and Fe. The strong interaction and electronic coupling between FeNi-LDH and V_2_C ensured prominent charge transfer. DOS and free-energy calculations further showed that this interfacial regulation not only improved charge-transfer capability, but also rendered the adsorption and desorption of reaction intermediates more balanced. The interfacial interaction, electronic coupling and charge transfer co-promoted the performance enhancement and improved the structural stability of the catalyst. Control experiments further show that V_2_C nanosheets exhibit negligible OER activity, confirming that the FeNi component supplies the principal catalytic phase. Together with the observed Ni^2+^-to-Ni^3+^ oxidation during activation and the Fe-doped NiOOH/O-terminated V_2_C theoretical model, these results support reconstructed FeNi oxyhydroxide as the probable working phase. V_2_C mainly serves as a conductive support and interfacial electronic regulator [70]. Hu et al. used Ti_3_C_2_T_x_-coated nickel foam as the support and loaded CoNi LDH onto it through electrodeposition, thereby obtaining the CoNi LDH/Ti_3_C_2_T_x_/NF electrode. The metallic conductivity, high hydrophilicity and electronegative surface of Ti_3_C_2_T_x_ jointly contributed to the construction of this structure. In an alkaline electrolyte, the CoNi LDH/Ti_3_C_2_T_x_/NF electrode exhibited an overpotential of 257.4 mV at 100 mA cm^−2^ and a Tafel slope of 68 mV dec^−1^. It also exhibited good durability, operating at 10 mA cm^−2^ for 50 h with slight current loss. Both experimental and theoretical results demonstrated electron transfer from CoNi LDH to Ti_3_C_2_T_x_. This charge transfer reduces the binding strength of reaction intermediates, thus regulating their adsorption in the OER process. The impedance results further showed that this heterostructure possessed a smaller charge-transfer resistance. Based on the general recognition that Co/Ni oxyhydroxides constitute the active OER phase, the authors represented the interface in their theoretical analysis using a CoNi-LDH monolayer supported on O-terminated Ti_3_C_2_O_2_. The calculations show electron transfer from CoNi-LDH to Ti_3_C_2_O_2_, a negative shift in the Co/Ni d-band center, and weakened binding of oxygen-containing intermediates. Thus, the Co/Ni centers in the LDH-derived working phase are the more probable catalytic sites, whereas Ti_3_C_2_T_x_ mainly acts as a conductive support and interfacial electronic regulator [71]. Yu et al. used Ti_3_AlC_2_ as the precursor and first synthesized Ti_3_C_2_ MXene by etching it in a LiF/HCl mixed system. After centrifugation and washing, the product was ultrasonically exfoliated in deionized water and the dark-green supernatant obtained after centrifugation was collected to yield a Ti_3_C_2_ MXene colloidal dispersion. Subsequently, Ni(NO_3_)_2_·6H_2_O, Fe(NO_3_)_3_·9H_2_O and urea were dissolved in degassed deionized water to form a precursor solution, while the Ti_3_C_2_ MXene colloid was dispersed in N-methylpyrrolidone to form a suspension. The two solutions were then mixed and refluxed under N_2_, followed by centrifugation, washing and freeze-drying to finally obtain the FeNi-LDH/Ti_3_C_2_-MXene nanohybrids. By adjusting the volume ratio of the two solutions, FeNi-LDH/Ti_3_C_2_-MXene samples with different FeNi-LDH contents could also be prepared. In 1.0 M KOH, the FeNi-LDH/Ti_3_C_2_-MXene catalyst containing about 80 wt% FeNi-LDH exhibited excellent OER performance, with an overpotential of about 298 mV at 10 mA cm^−2^ and a Tafel slope of 43 mV dec^−1^. Under identical conditions, its performance was superior to that of commercial RuO_2_, FeNi-LDH/rGO and FeNi-LDH + Ti_3_C_2_-MXene, whose η_10_ values were 358, 356 and 366 mV and whose Tafel slopes were 63, 60 and 64 mV dec^−1^, respectively. In addition, the optimized FeNi-LDH/Ti_3_C_2_-MXene catalyst maintained a working potential of about 1.56 V at 10 mA cm^−2^ for 12 h. This performance enhancement is associated with the strong interaction and pronounced charge transfer between FeNi-LDH and Ti_3_C_2_ MXene. XPS results showed that the Ni 2p and Fe 2p peaks shifted by 0.5–0.6 eV toward higher binding energies relative to pure FeNi-LDH, confirming a significant electronic modulation effect at the heterointerface. The study further pointed out that this interfacial effect facilitates the electrostatic attraction of more anionic intermediates into the FeNi-LDH lattice and accelerates the Ni^2+^/Ni^3+^,Ni^4+^ redox process. In the calculations, a heterointerface model was constructed by coupling a (001)-exposed Fe-doped NiOOH layer with O-terminated Ti_3_C_2_ MXene and the Ni:Fe ratio was set to 3:1, which is close to the experimentally measured value of about 2.5:1. The corresponding differential charge density analysis was used to characterize the interfacial electron redistribution after coupling, thereby supporting the conclusion that pronounced interfacial charge transfer occurs between FeNi-LDH and Ti_3_C_2_ MXene. In general, the regulation of interfacial charges not only affects the electron transport process but also influences the adsorption behavior of reaction intermediates. Pristine Ti_3_C_2_ exhibits negligible OER activity, confirming that FeNi-LDH supplies the catalytic phase. The Fe-doped NiOOH/O-terminated Ti_3_C_2_ model represents the probable working interface, in which the oxyhydroxide provides adsorption sites and Ti_3_C_2_ regulates interfacial charge density [72].

Furthermore, the role of the heterostructure can be further extended to the regulation of electronic-state distribution and oxygen activation. The enhancement of OER performance by the heterostructure results from the combined effects of interfacial charge transfer, adsorption regulation, electronic-state evolution and improved structural stability. Hao et al. grew CoFe-LDH in situ on the surface of Ti_3_C_2_ MXene, thereby constructing the CoFe-LDH/MXene composite catalyst. The hydroxyl-rich surface of Ti_3_C_2_ is favorable for the direct growth of CoFe-LDH. This process suppresses the aggregation of LDH and exposes more edge active sites. CoFe-LDH/MXene exhibited an overpotential of 319 mV at 10 mA cm^−2^ with a Tafel slope of 50 mV dec^−1^. In contrast, pure CoFe-LDH and Co(OH)_2_ required overpotentials of 352 and 370 mV to reach the same current density, respectively. When tested at a constant overpotential of 0.5 V, CoFe-LDH/MXene sustained a current density of about 2.7 mA cm^−2^ for 10 h. Ti_3_C_2_ provides a metallic and highly conductive substrate, thereby accelerating electron transport in the composite catalyst and offering more favorable electronic conditions for oxygen activation at the CoFe-LDH surface. DFT results further showed that interfacial coupling altered the electronic-state distribution. After hybridization, CoFe-LDH changed from a semiconductor with a band gap of about 1.65 eV to a system with more pronounced metallic features. In particular, the O 2p states were significantly enhanced near the Fermi level and further became distributed above the Fermi level. The authors attributed this change to the deeper insertion of the Co/Fe 3d bands into the O 2p band, which pushed the O 2p states to higher energy and generated localized O 2p holes, thereby benefiting interfacial charge transfer and oxygen activation [73]. Related MXene-OER studies further suggest that interfacial coupling may alter the surface state of the catalyst under anodic polarization. Therefore, MXene-containing heterostructures should be evaluated not only by their initial conductivity, but also by their reconstructed surface structure during OER [25,27,74,75,76,77]. Li et al. coupled the amorphous high-entropy borate FeCoNiMnBO_x_ with Ti_3_C_2_-MXene through a low-temperature liquid-phase reduction strategy to obtain the FeCoNiMnBO_x_/MXene composite catalyst. The catalyst denoted as HEBO_x_/MXene3, corresponding to a metal–salt–precursor-to-MXene ratio of 3:1, delivered an overpotential of 268 mV at 10 mA cm^−2^ in an alkaline medium and exhibited a Tafel slope of 39.8 mV dec^−1^. In addition, HEBO_x_/MXene3 operated at 10 mA cm^−2^ for 45 h with only a 1.07% increase in potential. Post-OER XPS further showed marked changes in the Fe, Co, Ni, and Mn valence states, disappearance of the B signal, and increased Ti–O species, indicating extensive reconstruction of the borate and partial oxidation of MXene. These results identify oxide or hydroxylated species as the working OER phase rather than the initial borate. The lamellar MXene in the composite suppressed the aggregation of FeCoNiMnBO_x_ and improved the utilization of active sites. In addition, the highly dispersed FeCoNiMnBO_x_ was observed to be amorphous, which enabled the exposure of a large number of active sites. The strong interfacial bonding between MXene and FeCoNiMnBO_x_ induced interfacial charge redistribution and accelerated charge transfer. This process improved the electrical conductivity of the catalyst while simultaneously promoting the oxidation process of the metal ions in FeCoNiMnBO_x_. In situ EIS further demonstrated that the interfacial synergistic coupling effect accelerated the kinetics of the OER [78].

**Figure 5:**
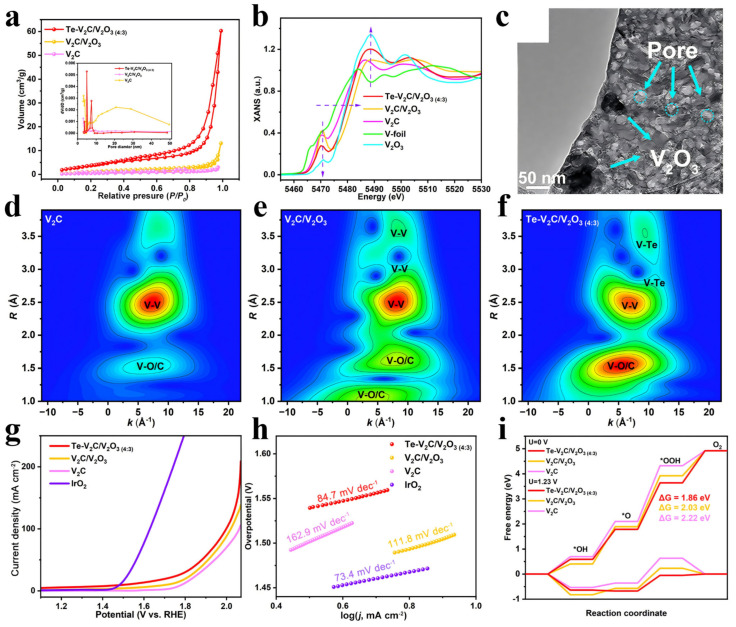
(**a**) N_2_ adsorption/desorption isotherms. Inset: pore size distribution curves of samples. (**b**) XANES spectra of V K-edge. Purple dashed arrows in (**b**) highlight the changes in the V K-edge XANES features. (**c**) TEM images of Te–V_2_C/V_2_O_3(4:3)_. Wavelet-transformed plots for the k^3^-weighted EXAFS signals of V K-edge of Cyan arrows and dashed circles in (**c**) indicate V_2_O_3_ domains and pores, respectively. (**d**) V_2_C, (**e**) V_2_C/V_2_O_3_, and (**f**) Te–V_2_C/V_2_O_3(4:3)_. (**g**) LSV curves of samples. (**h**) Tafel plots of samples. (**i**) Free energy diagram of OER for V_2_C, V_2_C/V_2_O_3_, and Te–V_2_C/V_2_O_3(4:3)_. In (**i**), * denotes an adsorbed intermediate at a catalytic site. Reprinted from Ref. [79].

Another interface-oriented example was reported by Wu et al., who combined a self-generated V_2_C/V_2_O_3_ phase boundary with supplementary Te doping and pore formation [79]. The catalyst was prepared by partially oxidizing and microetching V_2_C with H_2_O_2_, followed by freeze-drying and Te incorporation through thermal treatment. Partial oxidation generated V_2_O_3_ directly on V_2_C, thereby forming an intimate V_2_C/V_2_O_3_ interface rather than depositing an independent phase onto a prefabricated support. The N_2_ adsorption–desorption results show that Te–V_2_C/V_2_O_3(4:3)_ possesses a specific surface area of 15.4 m^2^ g^−1^, markedly higher than the 2.4 m^2^ g^−1^ of V_2_C, while the pore-size distribution confirms the formation of mesoporous channels (Figure 5a). The V K-edge XANES spectra indicate that Te incorporation changes the oxidation state and ligand-field environment of V (Figure 5b). TEM directly reveals pores and V_2_O_3_ nanoparticles distributed across the V_2_C framework (Figure 5c). Consistently, the wavelet-transformed EXAFS maps retain the V–V contribution while showing additional V–O/C and V–Te coordination features after interface formation and doping (Figure 5d–f). Te–V_2_C/V_2_O_3(4:3)_ requires an overpotential of 279.8 mV to achieve 10 mA cm^−2^, compared with 480.8 mV for V_2_C/V_2_O_3_ and 553.1 mV for V_2_C (Figure 5g). Its Tafel slope decreases to 84.7 mV dec^−1^, indicating accelerated OER kinetics (Figure 5h). The calculated free-energy diagrams identify *O-to-*OOH conversion as the potential-determining step and show that its energy requirement decreases from 2.22 eV on V_2_C and 2.03 eV on V_2_C/V_2_O_3_ to 1.86 eV on Te–V_2_C/V_2_O_3(4:3)_ (Figure 5i). Thus, the V_2_C/V_2_O_3_ interface provides the principal interfacial regulation, whereas Te doping modifies intermediate adsorption and microetching improves site accessibility. The overall water decomposition experiment results show that the Faraday efficiency of anode OER is 81.3%. Table 2 presents a comparison of representative interface-engineered MXene-based catalysts, illustrating the effects of interfacial coupling on their electrochemical performance and durability.

### 3.3. Morphology Engineering

This section focuses on the morphology and organization level, that is, how the active units are organized into catalytic structures that are more conducive to electrolyte infiltration, ion diffusion and gas escape through a three-dimensional skeleton, open pores, interlayer expansion, in-plane openings and hollow units. For the related work in arrayed or self-supporting electrodes, this paper regards it as the further development of morphology control under the condition of high current density, focusing on how three-dimensional arrays, hierarchical porous frameworks, loose epitaxial nanosheets, interlayer open spaces and outer surface open units can continue to improve electrolyte supply, shorten ion transport paths, promote bubble desorption and maintain the structural integrity and channel accessibility of catalytic layers under high-current-density conditions; related MXene-OER studies have further extended this morphology-oriented design to self-supported electrodes, MXene/NF frameworks, vertically aligned MXene/LDH structures and folded MXene nanosheets [80,81,82,83,84,85,86,87].

**Figure 6:**
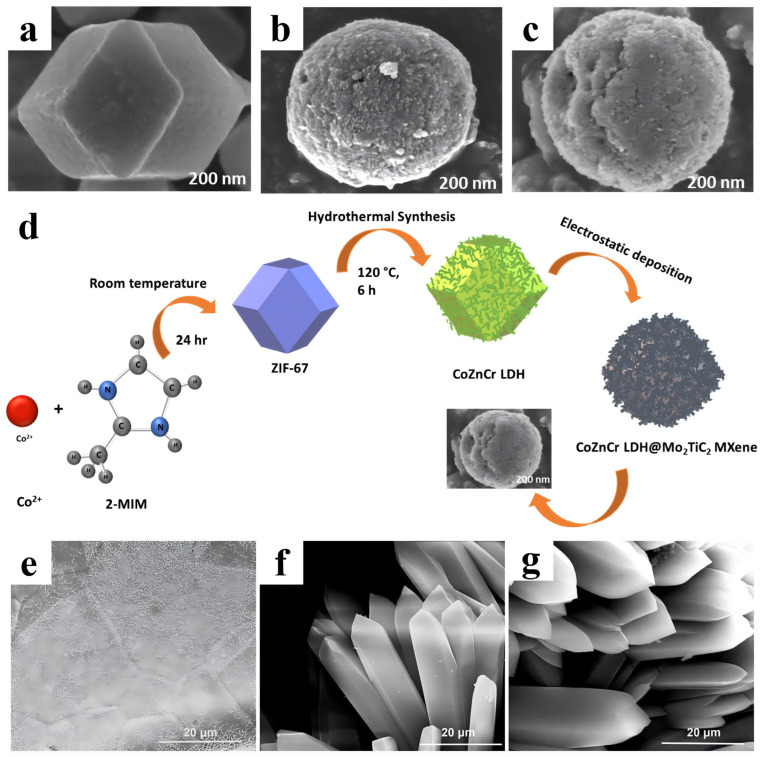
(**a**–**c**) SEM image of as-prepared ZIF-67, CoZnCr and CoZnCr@Mo_2_TiC_2_. (**d**) Schematic illustration of the synthesis process of CoZnCr@MXene. In (**d**), red, gray, blue, and dark-gray spheres represent Co, C, N, and H, respectively, whereas orange arrows indicate the synthesis sequence. Reprinted from Ref. [88]. Copyright 2025 The Royal Society of Chemistry. Published by the Royal Society of Chemistry. (**e**–**g**) SEM images: MXene/NF, CoNi_0.04_-MOF/MXene/NF, Co-MOF-74/MXene/NF. Reprinted from Ref. [89].

One morphology route begins with dodecahedral ZIF-67. The initially smooth and well-defined polyhedral precursor is approximately micrometer-sized (Figure 6a). Hydrothermal conversion largely preserves the particle outline while covering its surface with clustered CoZnCr-LDH nanosheets (Figure 6b). Subsequent assembly with Mo_2_TiC_2_ produces composite particles with a rougher, nanoparticle-decorated exterior (Figure 6c). The corresponding hydrothermal conversion and electrostatic deposition processes are summarized in the synthesis scheme (Figure 6d). Complementary HRTEM analysis in the original study further confirms the formation of a hollow nanocage, whose internal cavities facilitate electrolyte penetration and increase active-site accessibility. DFT calculations identify Cr as a favorable adsorption site and *O-to-*OOH conversion as the potential-determining step. Supporting calculations show that the theoretical OER overpotential decreases from 2.99 V for free-standing CoZnCr to 2.69 V for the F-terminated CoZnCr@Mo_2_TiC_2_F_2_ model, corresponding to a reduction of 0.30 V. Experimentally, CoZnCr@Mo_2_TiC_2_ requires an overpotential of 20 mV to reach 10 mA cm^−2^ in 1.0 M KOH and exhibits a reported Tafel slope of 47 mV dec^−1^. Direct O_2_ quantification in the alkaline-seawater anion-exchange-membrane electrolyzer gives a Faradaic efficiency of 99%, and stable operation was maintained for 16.8 days at 200 mA cm^−2^ [88].

Yu et al. prepared CoNi_0.04_-MOF-74/MXene/NF through MXene deposition on nickel foam followed by the hydrothermal growth of Ni-doped Co-MOF-74 [89]. The initial MXene coating is evenly distributed over the nickel-foam skeleton (Figure 6e). Regular hexagonal CoNi_0.04_-MOF-74 microrods with smooth surfaces and an average diameter of approximately 10 μm grow on the MXene-covered substrate (Figure 6f). Compared with the broader undoped Co-MOF-74 crystals (Figure 6g), Ni incorporation promotes the formation of thinner and longer microrods. The resulting electrode requires 256 mV at 100 mA cm^−2^ and exhibits a Tafel slope of 40.21 mV dec^−1^. After 1000 CV cycles, the microrod framework is retained, while HRTEM and SAED identify Co(OH)_2_, CoOOH, and Ni(OH)_2_ species. Post-OER XPS shows that the Co^3+^/Co^2+^ and Ni^3+^/Ni^2+^ ratios increase from 2.47 to 3.62 and from 0.34 to 0.54, respectively, supporting the formation of hydroxide–oxyhydroxide working species. Meanwhile, the disappearance of Ti–C and increase in Ti–O indicate partial oxidation of the MXene phase. Gas quantification in the overall electrolyzer gives a Faradaic efficiency of approximately 97%.

Li et al. first coated exfoliated Ti_3_C_2_T_x_ onto the surface of nickel foam to form an MXene/NF substrate and then carried out the one-pot hydrothermal growth process in a solution containing Co, Fe and phosphomolybdic acid precursors so that FeCo-LDH and P-MoO_3_ could be coupled in situ on the substrate surface. Ultimately, a three-dimensional porous celosia-like heterostructure was constructed. Morphological characterization showed that initial Ti_3_C_2_ MXene exhibited a clear multilayered accordion-like structure. After the above synthesis steps, sea urchin-like FeCo-LDH nanowires grew evenly and densely on the MXene/NF scaffold, forming an open three-dimensional framework with P-MoO_3_. This three-dimensional porous structure is beneficial to gas adsorption–desorption and effective mass transfer. For OER, this catalyst delivered an overpotential of 179 mV at 10 mA cm^−2^, with a Tafel slope of 40.44 mV dec^−1^ and a C_dl_ value of 9 mF cm^−2^. Quantitative gas collection gave H_2_ and O_2_ production rates of approximately 0.67 and 0.33 mmol h^−1^, respectively, with an O_2_ Faradaic efficiency of 96.30%, confirming that the anodic current predominantly originated from oxygen evolution. Post-OER XPS additionally revealed the formation of CoOOH. In terms of stability, P-MoO_3_ FCL MXene/NF showed almost no difference in current density before and after 1000 CV cycles while its structure remained relatively intact. Moreover, after 42 h of continuous stability testing, it still retained 98.27% of the current density [90].

Sheng et al. first used the Ti_3_AlC_2_ phase precursor (Ti_3_AlC_2_ MAX) as the precursor and synthesized few-layer Ti_3_C_2_T_x_ MXene through a minimally intensive layer delamination (MILD) method. After delamination in water, the obtained Ti_3_C_2_T_x_ nanosheets were separated by a gravity-based centrifugation method into MXene with a high O-termination ratio (HOMX) and MXene with a low O-termination ratio (LOMX). Subsequently, a series of CoFeLDH-Ti_3_C_2_T_x_ heterostructures were prepared by an in situ growth method with a fixed weight percentage of HOMX or LOMX as a template under environmental conditions, in which the feed ratios of Co:Fe atoms were 2:1, 3:1, 4:1 and 6:1. As a control, pure CoFeLDH nanosheets were synthesized under the same reaction conditions and at the same Co:Fe feeding ratios without adding Ti_3_C_2_T_x_. TEM showed that Co_4_Fe_1_-LOMX exhibited a house-of-cards assembly of two-dimensional nanosheets. XRD results further demonstrated that the introduction of Ti_3_C_2_T_x_ significantly reduced the domain size of CoFe-LDH and the domain size of Co_3_Fe_1_ and Co_4_Fe_1_ on LOMX decreased by about 70%. Co_4_Fe_1_-LOMX delivered an overpotential of 301 mV at 10 mA cm^−2^, with a Tafel slope of 43 mV dec^−1^ and a C_dl_ value of 0.618 mF cm^−2^. Additionally, it was reported that the overpotential decayed by 0.1% after 200 h of testing. Researchers explained that the growth guided by MXene produced small domain CoFeLDH nanosheets in the house-of-cards structure, which inhibited Ti_3_C_2_T_x_ re-accumulation and exposed more active sites. Together with the decrease in the number of layers along the (003) direction, this structure contributed to the long-term OER durability of the catalyst [91].

If three-dimensional frameworks are regarded as a way of organizing the external space, then interlayer expansion and the introduction of in-plane openings directly regulate the internal reaction space of MXene. Zhou et al. constructed porous V_2_C-MXene with lattice tensile strain and microscopic pores through rapid liquid-nitrogen freezing followed by hydrothermal treatment. The study showed that the microscopic pores could optimize the ion-transport pathway and shorten the migration distance, whereas the lattice tensile strain widened the interlayer spacing and accelerated ion-transport kinetics, ultimately enabling the material to deliver an OER overpotential of 269 mV at 10 mA cm^−2^. In addition, TS(24)-P(50)-V_2_C showed no obvious decay in current density after working for 24 h. Here, 24 in TS(24)-P(50)-V_2_C denotes the hydrothermal treatment time, whereas 50 denotes the mass of V_2_C-MXene used to prepare the porous precursor [92]. Qiu et al. proposed a hydrothermal-assisted strategy to construct stable bilayer-H_2_O-pillared MXene. This strategy facilitated the entry of metal precursor ions into the MXene interlayers and enabled in situ growth within the regulated interlayer/interfacial environment, ultimately forming NiFe/MX-HT. The study showed that bilayer-H_2_O pillaring significantly enlarged the MXene interlayer spacing and was associated with a higher interlayer ion concentration. Further, the researchers cited previous research results: in the double-layer H_2_O structure, the diffusion speed of substances is more than ten times faster than that in the single-layer structure. Electrochemical measurements showed that NiFe/MX-HT required only 230 mV to reach 10 mA cm^−2^ in 1 m KOH. Moreover, NiFe/MX-HT showed a potential increase of only 29 mV over 220 h at 10 mA cm^−2^ [93]. Shen et al. advanced structural regulation to in-plane hole engineering and constructed an LDH/H–Ti_3_C_2_T_x_ architecture in which holey Ti_3_C_2_T_x_ was intimately coupled with ultrathin Ni–Fe LDHs. In electrochemical measurements, the optimized LDH(60%)/H–Ti_3_C_2_T_x_ exhibited an operating overpotential of 310 mV at 50 mA cm^−2^ and a Tafel slope of 47 mV dec^−1^, indicating that this architecture still maintained fast OER kinetics at medium-to-high current densities. FE-SEM observations showed that H–Ti_3_C_2_T_x_ exhibited a lamellar nanosheet morphology prior to in-plane hole formation. The study showed that a large number of in-plane holes with sizes within 15–25 nm were generated on the surface of H–Ti_3_C_2_T_x_, after which ultrathin hexagonal Ni–Fe LDH nanolayers were uniformly distributed over its surface to form a porous 2D/2D heterojunction with open holes. High-resolution transmission electron microscopy (HRTEM) revealed lattice spacings of 0.18 nm for Ti_3_C_2_T_x_ and 0.25 nm for Ni–Fe LDHs, corresponding to the (211) plane of Ti_3_C_2_T_x_ and the (012) plane of Ni–Fe LDHs, respectively, indicating that both the holey MXene framework and the LDH nanolayers retained their original crystal structures after assembly. More importantly, N_2_ adsorption–desorption measurements showed that LDH/H–Ti_3_C_2_T_x_ possessed a specific surface area of 55.4 m^2^ g^−1^, which was significantly higher than that of LDH/Ti_3_C_2_T_x_ at 26.1 m^2^ g^−1^ and Ti_3_C_2_T_x_ at 13.2 m^2^ g^−1^. In-plane holes and ultra-thin active nano-layers together transform the mass transfer mode of a 2D layered structure from the mode dominated by interlayer diffusion to a more open mode involving in-plane channels and interlayer spaces. The increased specific surface area and lower charge transfer resistance further showed that in-plane pore engineering not only improves the accessibility of interlayer catalytic sites, but also helps to improve the overall mass transfer performance of 2D layered catalysts [94].

The above studies mainly addressed the accessibility of active sites within the nanosheets and between adjacent layers, whereas hollow units, hierarchical clustered structures and arrayed growth further optimize the catalyst toward an open outer-surface architecture. Zhou et al. anchored MOF-derived hollow CoV_2_O_6_ nanocubes onto lattice tensile-strained V_2_CT_x_ MXene through an ion-exchange process followed by liquid-nitrogen quenching treatment. The results show that CoV_2_O_6_ not only acts as an intercalation unit to inhibit the re-stacking of MXene nanosheets, but also provides a large active surface area and promotes electrolyte penetration and electron and ion transfer due to its hollow structure. As a result, the obtained material exhibited an OER overpotential of 235.0 mV at 10 mA cm^−2^ [95]. Li et al. constructed a grass-like ZnCoCH@Ti_3_C_2_T_x_ heterostructure by in situ solvothermal growth of grass-like ZnCoCH on the surface of Ti_3_C_2_T_x_ and the resulting catalyst delivered an overpotential of 280 mV at 10 mA cm^−2^ in 1.0 M KOH, with a Tafel slope of 46.2 mV dec^−1^. Contrary to the serious agglomeration of ZnCoCH in the absence of MXene, ZnCoCH in the composites grows uniformly and has a grass-like morphology on the surface and edges of Ti_3_C_2_T_x_. As the growth substrate, MXene reduced the agglomeration of ZnCoCH and exposed more active sites. This shows that the open framework and grass-like surface morphology provided by MXene are beneficial to the dispersion of active components and the exposure of active sites. Together with enhanced conductivity, interfacial charge redistribution, and oxygen-vacancy-related activation, these features promote OER performance [96].

On this basis, morphology engineering has been further extended to self-supporting array electrodes, where MXene-containing interlayers or coatings can strengthen the contact between the active phase and the current collector while helping to maintain electrolyte supply and gas release at higher current densities [88,97,98,99,100]. Wang et al. constructed a CoNi-LDH/MXene@NiMoO_4_ multistage nanoarray electrode. NiMoO_4_ first formed a uniform nanorod array on NF; MXene was introduced as an intermediate layer covering the outer surface of the nanorods and CoNi-LDH was then further grown by electrodeposition as vertically arranged nanosheets. This process ultimately generated a multistage architecture composed of nanorods, an intermediate MXene layer and vertically arranged nanosheets. The introduction of MXene inhibited the spherical agglomeration and the stacking of MXene sheets, which often occurred in CoNi-LDH, so that the nanosheets were arranged on the surface of nanorods in a looser epitaxial manner, and at the same time, the spacing between nanorods was enlarged, providing more sufficient open channels and increasing the contact area between electrolyte and nanorod substrate. In terms of performance, the optimized CoNi-LDH/MXene@NiMoO_4_ electrode delivered an OER overpotential of 222 mV at 100 mA cm^−2^ with a Tafel slope of 84.2 mV dec^−1^ and remained stable for 120 h. In a chronoamperometry test at 20 mA cm^−2^, CoNi-LDH/MXene@NiMoO_4_ maintained stable OER operation for 50 h. Even under 3 M KOH at 300 mA cm^−2^, it still worked stably for 20 h and after prolonged OER stability tests, both the CoNi-LDH nanosheets and the overall nanorod-array morphology remained intact. This array architecture simultaneously features widened nanorod spacing, vertically arranged nanosheets and an open interfacial configuration and these structural factors may jointly favor electrolyte wetting, ion transport and morphological retention under high-current operation [101].

The CoFe-P@MXene/NF constructed by Guo et al. further indicates that when the above porous architecture is introduced into a nickel foam-supported electrode, its advantages in open mass transport can still be manifested at high current density. In this work, a three-dimensional porous catalytic layer featuring multiple heterojunctions of Co_2_P/Ti_3_C_2_T_x_, Fe_2_P/Ti_3_C_2_T_x_ and Co_2_P/Fe_2_P was fabricated through strong electrostatic self-assembly, electrodeposition and low-temperature phosphorization. In this system, MXene suppressed the aggregation of transition-metal phosphides and induced the construction of a three-dimensional porous framework. The porous structure, together with the hydrophilicity and conductivity of MXene, jointly promoted electrolyte penetration and the release of gaseous products. In 1 M KOH, this catalyst required only 215 mV to reach 20 mA cm^−2^ and needed only 328 mV at 1000 mA cm^−2^. Direct gas collection by the water-displacement method gave an OER Faradaic efficiency above 97% at 100 mA cm^−2^ and above 90% at 500 mA cm^−2^, verifying efficient oxygen production even under high-current operation. Post-OER TEM and XPS further showed that part of Co_2_P and Fe_2_P was oxidatively reconstructed into amorphous CoOOH and FeOOH, identifying these oxyhydroxides as the working surface phases. In addition, stability tests showed that it could work stably for 100 h at 100 mA cm^−2^ in 6 M KOH at 60 °C. Chronoamperometry further showed that there was only a slight change after 80 h at 20 mA cm^−2^. Multi-step chronopotentiometric testing further showed that over the range of 10–200 mA cm^−2^, the potential responded rapidly to changes in current density and each stepwise plateau remained stable. These results suggest that, under higher current-density conditions, the open porous structure may still be beneficial for OH^−^ supply and the detachment of gaseous products [102].

Curvature represents another aspect of morphology engineering that extends MXene regulation beyond flat two-dimensional sheets. First-principles calculations showed that MXene nanoribbons with chemically asymmetric surfaces can spontaneously bend to relieve the imbalance between their two surfaces, with the curvature increasing as the ribbon width decreases. The resulting bending, edge reconstruction, and transition-metal hybridization can modify edge states and the electronic structure [103]. Calculations on functionalized Sc_2_C nanotubes further demonstrated that curvature effects are strongly dependent on surface termination. With increasing curvature, the band gap decreased for Sc_2_CH_2_ nanotubes but increased for Sc_2_C(OH)_2_ nanotubes, indicating that curvature cannot be considered independently of surface chemistry [104]. The experimental feasibility of regulating MXene curvature was demonstrated by Vaughn et al., who employed different p-phosphonic acid calixarenes during the delamination of Ti_2_C to selectively obtain plates, crumpled sheets, spherical particles, and scrolls. In particular, PCX8 produced MXene scrolls with diameters of approximately 0.5–0.9 μm [105]. From an OER perspective, moderate curvature may suppress face-to-face restacking, expose additional edge sites, create open electrolyte-transport pathways, and alter local coordination through bending-induced strain. However, excessive curvature may also cause severe structural distortion or reduce the stability of the conductive MXene framework. Because the above studies primarily established the structural and electronic consequences of curvature rather than directly measuring OER, its catalytic contribution should be evaluated using compositionally identical flat and curved samples, together with overpotential, Tafel slope, stability, ECSA-normalized activity, oxygen quantification, Faradaic efficiency, operando characterization, and post-OER structural analysis.

In summary, the morphological modifications in MXene-based OER systems involve the opening of the outer surface, the reorganization of interlayer spaces, the creation of in-plane openings and the transition from porous structures to self-supporting structures. These changes collectively affect electrolyte wetting, ion diffusion and gas release processes. For a clearer comparison of how different morphological architectures influence active-site accessibility and mass transport, the principal OER performance parameters are provided in Table 3.

### 3.4. Composite Engineering

Following the progression from lower-dimensional local regulation to higher-dimensional morphological regulation, composite engineering represents a more macroscopic and system-level modification strategy. In contrast to the preceding approaches, which generally focus on a dominant factor such as the local electronic structure, a specific heterointerface or a particular morphological feature, composite engineering treats the entire multicomponent catalyst as the object of regulation. Different components are selected to compensate for their respective limitations and perform complementary functions. Their coordinated integration can consequently produce higher activity, faster reaction kinetics or greater stability than catalysts constructed through an individual modification route. This enhancement is generally achieved through the simultaneous regulation of several underlying physicochemical factors, including electronic-state distribution, adsorption of oxygen-containing intermediates, interfacial charge transfer, electrical conductivity, active-phase dispersion, accessible surface area, electrolyte wettability, ion diffusion, bubble release and structural retention under anodic conditions.

The most typical MXene-based composites involve coupling MXenes with carbon materials, including graphene, reduced graphene oxide, carbon nanotubes and N-doped carbon. These carbon components mainly construct continuous conductive networks, suppress the restacking of MXene nanosheets and the aggregation of active particles and improve the accessibility of the catalyst surface. Another MXene-based composite combines MXenes with other chemicals, including hydroxides, oxides, chalcogenides, phosphides and fluorides. Its boundary with heterointerface engineering is relatively blurred because the introduction of another chemical phase inevitably generates interfacial interactions. Nevertheless, the two strategies differ in their main analytical focus. Heterointerface engineering primarily emphasizes the local charge redistribution and bonding interaction at a specific phase boundary, whereas composite engineering focuses on the cooperative functions of all components within the entire catalyst architecture. In most of these composites, the secondary chemical phase provides the principal OER-active sites, while the MXene phase serves as a conductive framework, nucleation substrate, electronic regulator, dispersion medium or structural stabilizer. Upon introducing the conductive MXene phase, the role of the hybrid structure is mainly manifested in enhanced electron-transport efficiency and reduced interfacial charge-transfer resistance. Furthermore, a lot of research has further coupled the MXene phase with LDH to strengthen the electrical contact between the active phase and conductive frame [107,108,109,110,111]. Zhu et al. used Ti_3_C_2_T_x_/reduced graphene oxide (rGO) as a hybrid support and deposited NiFe-LDH onto it via a chemical deposition method, thereby obtaining the NiFe-LDH/Ti_3_C_2_T_x_-rGO catalyst. Ti_3_C_2_T_x_-rGO was formed by the self-assembly of positively charged CTAB-rGO and negatively charged Ti_3_C_2_T_x_. Herein, Ti_3_C_2_T_x_-rGO was introduced into the hybrid support system mainly as a conductive phase. NiFe-LDH was subsequently deposited on the surface of this support. Morphological characterization showed that NiFe-LDH grew as nanoflakes on the Ti_3_C_2_T_x_-rGO surface. This catalyst exhibited superior OER activity in 1.0 M KOH. NiFe-LDH/Ti_3_C_2_T_x_-rGO delivered an overpotential of 235 mV at 10 mA cm^−2^, lower than the 271 mV of NiFe-LDH/rGO and its Tafel slope was 40 mV dec^−1^, markedly smaller than those of NiFe-LDH/rGO (56 mV dec^−1^) and pure NiFe-LDH (80 mV dec^−1^). EIS results showed that NiFe-LDH/Ti_3_C_2_T_x_-rGO exhibited the lowest charge-transfer resistance (R_ct_) of 4.2 Ω among all samples. In contrast, the corresponding values for NiFe-LDH/rGO, pure NiFe-LDH and RuO_2_ were 8, 11.6 and 52 Ω, respectively. The C_dl_ results also indicated that this hybrid system possessed a larger electrochemically active surface area (ECSA), with C_dl_ values of 0.26, 0.21 and 0.08 μF cm^−2^ for NiFe-LDH/Ti_3_C_2_T_x_-rGO, NiFe-LDH/rGO and pure NiFe-LDH, respectively. Chronopotentiometric measurements further showed that NiFe-LDH/Ti_3_C_2_T_x_-rGO maintained stable operation at a current density of 10 mA cm^−2^ for 12 h with an increase of 6 mV in potential. This performance is closely related to interfacial chemical bonding, spontaneous charge transfer and the increased exposure of active sites. XPS results showed that the characteristic Ni 2p and Fe 2p peaks shifted by 0.4 and 0.3 eV, respectively, toward higher binding energy. The authors attributed this change to the chemical interaction between NiFe-LDH and Ti_3_C_2_T_x_-rGO, together with the accompanying spontaneous charge transfer. This process renders the Ni and Fe active centers more positively charged, thereby facilitating the electrostatic attraction of anionic intermediates and accelerating the redox process of NiFe-LDH during OER. The study also pointed out that the introduction of MXene effectively improved the dispersion of NiFe-LDH, increased the number of exposed active sites and markedly enhanced the charge-transfer capability and structural stability of the system [112]. De et al. used a Ti_3_C_2_T_x_/NH_2_-rGO precursor, denoted as MNG, as the substrate to fabricate the MNG-MoSn_2_Se_4_ composite catalyst. The precursor can provide a conducting matrix for the metal selenides and suppress their agglomeration tendency. SEM further showed that MoSn_2_Se_4_ was uniformly distributed over the MNG sheets, with particles intercalated into the interlayer spaces and coated on the surfaces of the MNG nanosheets. This catalyst exhibited superior OER activity in 1 M KOH. MNG-MoSn_2_Se_4_ exhibited an overpotential of 50 mV at 10 mA cm^−2^, lower than those of MNG-MoSe_2_ (240 mV), MNG-SnSe (200 mV), MNG (712 mV), MXene (718 mV) and NH_2_-rGO (717 mV). Its Tafel slope was 55.9 mV dec^−1^, which was smaller than those of MNG-MoSe_2_ (87.3 mV dec^−1^), MNG-SnSe (97.7 mV dec^−1^), MNG (136.8 mV dec^−1^), MXene (161.8 mV dec^−1^) and NH_2_-rGO (166.8 mV dec^−1^). The Nyquist plots further indicated that MNG-MoSn_2_Se_4_ exhibited lower solution resistance and charge-transfer resistance, together with superior capacitive behavior. The conductive network constructed by NH_2_-rGO and MXene facilitated electron transport and accelerated the reaction kinetics during OER. This improvement is mainly attributed to the conductive substrate and the inhibition of agglomeration. MNG-MoSn_2_Se_4_ composites show low diffusion resistance, which is beneficial to electrochemical transport. At the same time, the two-dimensional composite substrate formed by NH_2_-rGO and MXene effectively inhibits the agglomeration tendency of metal selenides, thus helping to retain accessible active sites [113].

The above systems illustrate the most typical form of composite engineering involving MXene and nanocarbon materials. In the NiFe-LDH/Ti_3_C_2_T_x_-rGO catalyst, rGO and MXene jointly construct the conductive support, whereas NiFe-LDH provides the main OER-active centers. In the MNG-MoSn_2_Se_4_ catalyst, NH_2_-rGO and MXene form a two-dimensional conductive and dispersion framework for the metal selenide. Therefore, the performance enhancement is not produced by a single interfacial effect. It results from the combined improvement of active-phase dispersion, electrical transport, exposed surface area and structural retention. Nevertheless, for the MNG-MoSn_2_Se_4_ system, the reported polarization, impedance and stability measurements did not include Faradaic efficiency or quantitative O_2_ measurements. Because metal selenides may undergo anodic oxidation, these electrochemical results demonstrate enhanced anodic activity but cannot independently confirm the quantitative conversion of current into oxygen.

**Figure 7:**
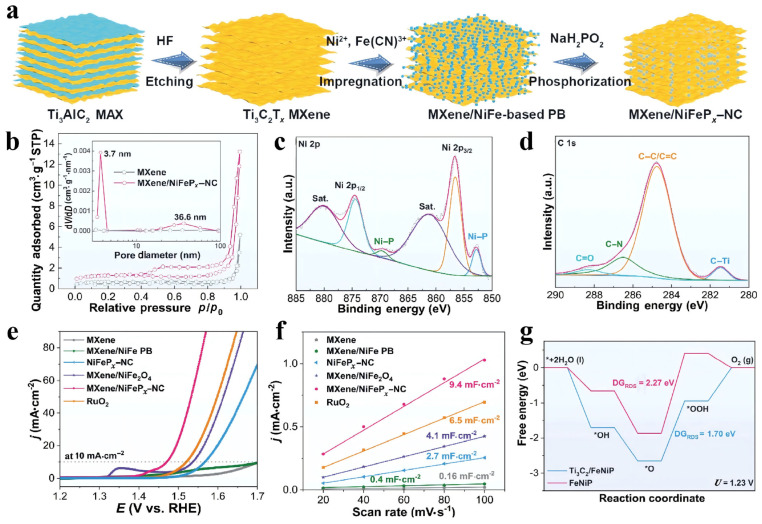
(**a**) Schematic illustration of synthesis of MXene/NiFeP_x_–NC electrocatalyst. (**b**) N_2_ adsorption–desorption isotherms of MXene and MXene/NiFeP_x_–NC heterostructure, wherein STP refers to standard temperature and pressure. (**c**,**d**) XPS spectra of Ni 2p and C 1s for the MXene/NiFeP_x_–NC heterostructure, wherein “Sat.” refers to satellite. (**e**) OER polarization curves and (**f**) C_dl_ of MXene, MXene/NiFe PB, NiFeP_x_–NC, MXene/NiFe_2_O_4_, MXene/NiFeP_x_–NC, and RuO_2_ catalysts. (**g**) Calculated OER free-energy diagrams of FeNiP (111) and MXene/NiFeP_x_ where ΔG_RDS_ refers to the changes in Gibbs reaction free energy for the rate-determining step (RDS). In (**g**), * denotes an adsorbed OER intermediate at a catalytic site. The colored curves and fitted components in (**b**–**g**) correspond to the samples or species identified by the respective in-panel legends and labels. Reprinted from Ref. [114].

Composite engineering represents the highest-dimensional regulation considered here because several functional components are integrated to improve different limiting factors simultaneously. Zeng et al. prepared a honeycomb-like MXene/NiFeP_x_–NC composite containing a NiFeP_x_ active phase, an N-doped carbon protective component, and a conductive Ti_3_C_2_T_x_ framework [114]. As illustrated in Figure 7a, Ti_3_C_2_T_x_ was first obtained from Ti_3_AlC_2_ and impregnated with Ni and Fe cyanide precursors. Subsequent phosphorization converted the precursor into NiFeP_x_ nanoparticles encapsulated by N-doped carbon and anchored throughout the MXene framework. In the absence of MXene, NiFeP_x_–NC particles exhibited severe agglomeration. By contrast, the MXene-containing composite retained NiFeP_x_ nanoparticles with sizes of approximately 10–30 nm and an NC coating of approximately 2 nm, indicating that MXene acted as both a conductive support and a spatial-confinement framework. The NC layer further improved conductivity and protected the dispersed particles. The N_2_ adsorption–desorption curves and pore-size distribution confirm that the final composite possesses a more open multichannel structure than pristine MXene (Figure 7b). Its specific surface area increases from 2.4 to 4.5 m^2^ g^−1^, together with the appearance of abundant mesopores. The Ni 2p spectrum confirms Ni–P bonding (Figure 7c), while the C 1s spectrum contains C–Ti, C–C/C=C, C–N, and C=O components, supporting the coexistence of MXene and N-doped carbon (Figure 7d). MXene/NiFeP_x_–NC requires only 240 mV at 10 mA cm^−2^, compared with 337 mV for NiFeP_x_–NC, 305 mV for MXene/NiFe_2_O_4_, and 290 mV for RuO_2_ under the reported conditions (Figure 7e). Its C_dl_ reaches 9.4 mF cm^−2^, indicating a larger electrochemically accessible surface than the individual and control components (Figure 7f). DFT calculations provided further information concerning the role of the MXene phase and the possible OER pathway. Bader charge analysis showed that approximately 2.94 electrons were transferred from Ti_3_C_2_ MXene to the NiFeP_x_ active phase. The calculated H_2_O adsorption energy changed from −1.71 eV on NiFeP_x_ to −0.505 eV on MXene/NiFeP_x_, indicating that the introduction of MXene produced more suitable water adsorption and activation. The calculated OER pathway involved the successive formation of *OH, *O and *OOH, with *OOH formation identified as the rate-determining step. Coupling with MXene decreased the corresponding free-energy barrier from 2.27 to 1.70 eV. These calculations support an adsorbate-mediated OER pathway and show that MXene regulates the adsorption energies of oxygen-containing intermediates. The material further exhibited an overpotential increase of only 7 mV after 2000 cycles and a current-density decrease of 3.3% after 30 h. Although the material contains heterointerfaces, its assignment to composite engineering is more appropriate because its performance depends on the coordinated functions of several components rather than a single phase boundary.

**Figure 8:**
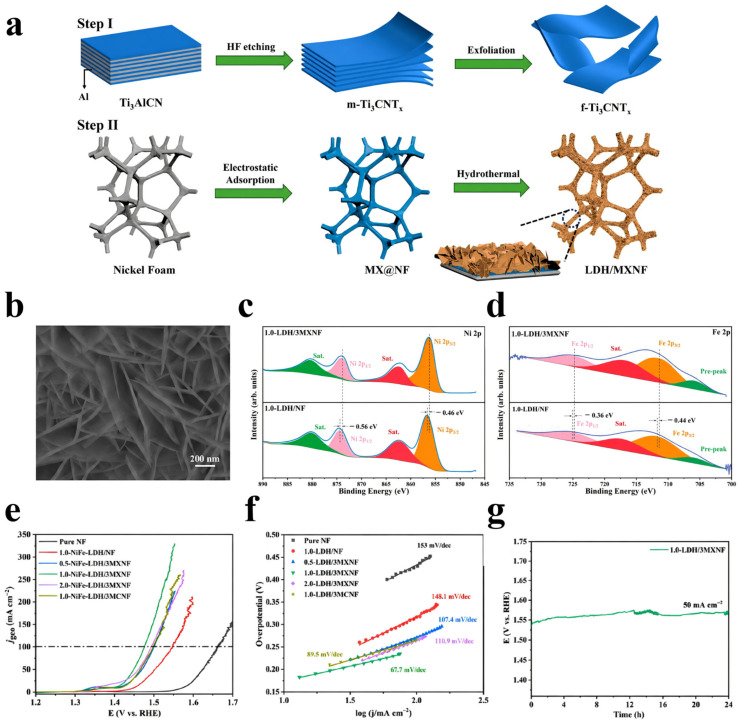
(**a**) Schematic synthetic route of LDH/MXNF self-standing electrode. The dashed circle identifies the local LDH/MXNF region highlighted in the enlarged inset. (**b**) SEM. (**c**) Ni 2p XPS spectra of 1.0-LDH/NF and 1.0-LDH/3MXNF; (**d**) Fe 2p XPS spectra of 1.0-LDH/NF and 1.0-LDH/3MXNF. The OER performance of the electrocatalysts in 1.0 M KOH. (**e**) LSV curves, (**f**) Tafel plots, and (**g**) long-term chronopotentiometric response at 50 mA cm^−2^. Reprinted from Ref. [81].

He et al. extended composite engineering from powder-level phase coupling to a self-standing electrode composed of NiFe-LDH, Ti_3_CNT_x_ MXene, and nickel foam [81]. As illustrated in Figure 8a, Ti_3_AlCN was first etched and exfoliated to obtain few-layer Ti_3_CNT_x_. The negatively charged MXene nanosheets were adsorbed onto nickel foam, after which NiFe-LDH was grown hydrothermally on the MXene-modified skeleton. The resulting structure integrates an OER-active LDH phase, a conductive Ti_3_CNT_x_ layer, and a porous macroscopic current collector. The SEM image shows densely interlaced NiFe-LDH nanosheets grown on the three-dimensional framework (Figure 8b). This vertical arrangement exposes LDH edges, preserves open electrolyte channels, and promotes O_2_-bubble release. Compared with NiFe-LDH grown directly on nickel foam, the Ni 2p and Fe 2p peaks of 1.0-LDH/3MXNF shift by approximately 0.2~0.6 eV toward lower binding energies (Figure 8c,d). These shifts indicate electron transfer from Ti_3_CNT_x_ to the Ni and Fe centers and demonstrate that MXene acts as an electronic bridge between the active LDH phase and nickel foam. The optimized 1.0-LDH/3MXNF electrode requires an overpotential of 247 mV to reach 100 mA cm^−2^ (Figure 8e) and exhibits the lowest Tafel slope of 67.7 mV dec^−1^ among the compared samples (Figure 8f). The electrode maintains an almost constant potential during 24 h operation at 50 mA cm^−2^ (Figure 8g). Control measurements showed that Ti_3_CNT_x_ alone possessed negligible OER activity, confirming that NiFe-LDH provides the principal active sites, whereas MXene improves electrical contact, active-phase integration, and electron transport. This case is therefore placed in composite engineering because its performance arises from the coordinated operation of the LDH active phase, MXene conductive layer, vertical nanosheet architecture, and macroscopic nickel-foam support.

Composite engineering with other chemical phases can further extend from powder catalysts to the coordinated construction of the entire electrode. He et al. prepared a self-standing Ti_3_CN/NiFe-LDH composite electrode on nickel foam. Ti_3_AlCN was first etched and exfoliated to obtain few-layer Ti_3_CN MXene. The negatively charged Ti_3_CN nanosheets were then adsorbed onto acid-treated nickel foam through electrostatic interactions, followed by the hydrothermal growth of NiFe-LDH nanoflakes. Oxygen-containing surface groups on Ti_3_CN adsorbed Ni^2+^ and Fe^3+^ and promoted the nucleation and crystallization of NiFe-LDH. As a result, vertically interlaced NiFe-LDH nanoflakes were formed on the three-dimensional MXene-modified nickel-foam framework. In this composite, NiFe-LDH served as the main OER-active phase, while Ti_3_CN provided a nucleation substrate and acted as an interfacial bridge between NiFe-LDH and nickel foam. The negligible OER activity of Ti_3_CN alone further indicated that the MXene phase mainly promoted the utilization of the active phase rather than acting as the principal active component. XPS results showed that the Ni 2p and Fe 2p binding energies of NiFe-LDH/Ti_3_CN shifted by approximately 0.2–0.6 eV toward lower values relative to NiFe-LDH/NF. The authors attributed these changes to electron transfer from Ti_3_CN to NiFe-LDH, which increased the electron density of the Ni and Fe species and confirmed strong coupling between the two components. The optimized 1.0-LDH/3MXNF electrode required an overpotential of 247 mV to reach 100 mA cm^−2^ and exhibited a Tafel slope of 67.7 mV dec^−1^. In comparison, 1.0-LDH/NF required an overpotential of 318 mV and exhibited a Tafel slope of 148.1 mV dec^−1^, whereas the Ti_3_C_2_T_x_-containing 1.0-LDH/3MCNF electrode required 270 mV and exhibited a Tafel slope of 89.5 mV dec^−1^. The Cdl value increased from 0.185 mF cm^−2^ for 1.0-LDH/NF to 0.396 mF cm^−2^ for 1.0-LDH/3MXNF, while the charge-transfer resistance decreased from 0.82 to 0.51 Ω. The electrode also maintained stable operation at 50 mA cm^−2^ for 24 h. Importantly, increasing the MXene content did not continuously improve the catalytic activity. Excessive MXene loading resulted in cracking, warping and partial detachment after hydrothermal treatment, thereby weakening the connection between NiFe-LDH and nickel foam and reducing the number of accessible sites. This result demonstrates that the advantage of composite engineering depends on the appropriate ratio and spatial organization of its components rather than their simple accumulation. The Tafel slope was interpreted as suggesting that a later stage of the multielectron-transfer process may be kinetically limiting. However, Tafel analysis alone cannot identify the OER pathway. Faradaic efficiency, quantitative O_2_ evolution and operando identification of the active phase were not reported; therefore, the available results mainly demonstrate improved charge transport, active-phase utilization and OER-associated electrochemical kinetics [81].

Xu et al. first grew Co(OH)F nanosheets on the MXene surface and then obtained the CoF_2_/MXene composite catalyst through subsequent low-temperature fluorination. This catalyst required an overpotential of 275 mV to reach 10 mA cm^−2^ and exhibited a Tafel slope of 50.7 mV dec^−1^. Post-OER TEM, XRD, and XPS showed that CoF_2_ was partially converted into CoOOH, accompanied by F leaching, demonstrating that the initial fluoride acted as a precatalyst. However, because no Faradaic efficiency or quantitative O_2_ measurement was reported, the contribution of precursor oxidation to the initial anodic current could not be independently excluded. The introduction of MXene modulated the d-band structure of Co and enhanced the adsorption of oxygen-containing intermediates. In addition, interfacial charge redistribution together with the high electronegativity of F promoted surface reconstruction, thereby facilitating the formation of highly active cobalt species. This change lowered the energy barrier of the rate-determining step. Thus, the improvement arises not only from enhanced conductivity, but also from the accelerated formation of the active phase [115]. The reconstruction of CoF_2_/MXene was supported by post-OER XRD, TEM and XPS measurements. New diffraction features associated with metal oxyhydroxides appeared after the stability test, and the initially deposited nanoparticles were transformed into nanosheets. Lattice fringes corresponding to CoOOH were identified, while the Co 2p_3/2_ peak shifted to a CoOOH-related position at 780.3 eV. These results indicate that the original CoF_2_ surface was converted into CoOOH species during OER and that the reconstructed CoOOH, rather than the initial fluoride surface, constituted the main working-state active phase. DFT calculations based on the reconstructed cobalt-hydroxide surface involved *OH, *O and *OOH intermediates and identified *OOH formation as the rate-determining step. The corresponding energy barrier decreased from 1.97 eV for CoF_2_ to 1.84 eV for CoF_2_/MXene. Thus, post-reaction characterization and theoretical calculations jointly support surface reconstruction and an adsorbate-mediated reaction pathway. Nevertheless, because the structural measurements were performed after OER and no Faradaic efficiency, quantitative O_2_ or operando measurements were reported, the real-time formation of the active phase and the oxygen-production selectivity still require further verification.

These examples show that composite engineering realizes the higher-dimensional integration of multiple modification effects. Its advantage does not originate merely from the coexistence of different materials, but from the coordinated assignment of their functions throughout the catalyst. The active chemical phase generally provides the main adsorption and conversion sites for OER intermediates, whereas MXene can simultaneously promote electron transport, regulate the electronic state of the active phase, guide nucleation, suppress agglomeration and maintain structural contact. Nanocarbon components can further construct conductive networks and protect dispersed particles, while macroscopic substrates provide mechanical support and open transport pathways. Consequently, composite engineering integrates electronic regulation, interfacial coupling, surface accessibility, mass transport and structural stability within a single catalytic architecture. Table 4 compares representative composite-engineered MXene-based catalysts and highlights the performance achieved through the coordinated integration of active phases, conductive components, and supporting structures.

## 4. Surface Restructuring of MXene-Based Catalysts

Importantly, “MXene-based catalyst” does not necessarily mean that the MXene phase itself is the active site of OER. Depending on the composition of the catalyst and its evolution under anodic polarization, MXenes can be used as a catalytic active phase, conductive carrier, precursor of oxidation skeleton, interfacial electron regulator or main body of atomic dispersion active center, etc. In many systems containing LDH, phosphide, sulfide, fluoride, and borate, transition metal components may be oxidized or reconstructed into high-valent metal hydroxyl oxides during OER, which constitute the actual or more likely working phase. In this case, MXenes mainly promote electron transport, inhibit aggregation, regulate interfacial charge redistribution, stabilize reconstructed species, and change intermediate adsorption.

In Section 3, it could be seen that the improvement of the catalytic performance of many MXene-based OER catalysts is closely related to the surface reconstruction (i.e., surface oxidation) of the catalysts during OER. However, the oxidation of MXene-based catalysts under anodic OER conditions should not be regarded as either entirely beneficial or entirely detrimental. Instead, it is necessary to distinguish the reconstruction of the catalytic phase from the oxidation of the MXene framework. Beneficial reconstruction generally refers to the conversion of transition-metal precursors into catalytically active high-valence oxides or oxyhydroxides, while the MXene retains its role as a conductive support and interfacial regulator. In contrast, destructive degradation occurs when oxidation progressively consumes the M–C framework of MXene, produces electrically insulating oxides, induces metal dissolution, or damages the layered conductive network.

The intrinsic susceptibility of Ti_3_C_2_T_x_ to electro-oxidation explains this narrow operating window. Single-entity measurements showed irreversible Ti_3_C_2_T_x_ oxidation above approximately 0.3 V vs. SCE, with the oxidation extent increasing with applied potential. XPS detected Ti loss that was attributed to soluble Ti species, while Raman spectroscopy showed weakened Ti_3_C_2_T_x_ bands, oxidation-induced holes and defects, and the formation of amorphous carbon. Although this potential was obtained in an acidic model electrolyte and should not be treated as a universal OER threshold, the results demonstrate that prolonged anodic polarization can progressively consume the Ti–C framework [116].

Furthermore, Selvam et al. investigated the post-OER transformation of CoP/MXene and Co_7_Se_8_/MXene using XPS, HR-TEM, EDX, ICP-OES, and EIS. After 10 h of OER operation, the disappearance of Co–P and Co–Se signals and the formation of new Co–O features demonstrated that both precursors were reconstructed into surface Co oxyhydroxide. However, the consequences of anion oxidation were distinctly different. The PO_x_-enriched Co–OOH/CoP/MXene surface retained its catalytic activity, and its charge-transfer resistance only changed from 1.72 to 3.46 Ω. Conversely, SeO_x_ accumulated on the Co–OOH/Co_7_Se_8_/MXene surface, increasing the charge-transfer resistance from 3.35 to 16.2 Ω and markedly deteriorating the OER activity and stability. Therefore, the formation of an oxyhydroxide phase can be beneficial only when the accompanying oxidation products do not cover active sites or interrupt charge transport [117].

The synthesis route also determines whether active-phase reconstruction can proceed without destroying the MXene support. Kaplan et al. explicitly assigned the Co oxide/hydroxide phase as the active-site provider and Ti_3_C_2_T_x_ as the conductive component. During OER, CoO or Co(OH)_2_ was oxidized into higher-valence Co species. Meanwhile, the Ti–C signal completely disappeared from the post-OER spectra of the physically mixed composites but remained detectable in the chemically functionalized samples. Moreover, the Ti oxidation peak was observed in the physically mixed materials but was absent in the chemically functionalized composites. These results indicate that chemically anchored Co oxide/hydroxide can act as a protective surface layer, allowing reconstruction of the Co phase while partially suppressing electrochemical oxidation of Ti_3_C_2_T_x_ [118].

Nevertheless, increasing the MXene content does not necessarily improve the balance between destructive degradation of MXene and reconstruction of the active phase. Schröder et al. found that NiOₓ/1% Ti_3_C_2_T_x_ exhibited the optimum OER performance, whereas higher MXene loadings produced larger amounts of surface TiO_2_. Operando Raman spectroscopy identified β-NiOOH as the working OER phase and showed that it appeared at 1.50 V vs. RHE in NiO_x_/1%Ti_3_C_2_T_x_, compared with 1.55 V vs. RHE for pure NiO_x_. However, the 10%Ti_3_C_2_T_x_ composite exhibited the worst stability, as a large amount of Ti_3_C_2_T_x_ was oxidized to TiO_2_ during the OER process; the excess TiO_2_ hindered the electron transfer in the catalyst and could also cover the accessible Ni sites. The results of ICP-OES show that the dissolution of titanium to form TiO_2_ is more obvious when the content of MXene is high. Therefore, it is necessary to find the best addition amount of MXene to avoid producing too many catalytically inactive oxidation products and promote the reconstruction of catalytically active phases [119].

Furthermore, beneficial reconstruction of active phases may even be suppressed by an unsuitable MXene. In Ti_3_C_2_T_x_/CoNi-MOF nanosheets, the incorporation of MXene improved electrical conductivity but decreased the intrinsic OER activity of the Co/Ni sites by more than 70%. Post-OER XPS showed that Co^2+^ and Ni^2+^ were readily oxidized into Co^3+^ and NiOOH in the unsupported CoNi-MOF, whereas these transformations were suppressed in the MXene-containing composite. This behavior was attributed to unfavorable electron donation from Ti_3_C_2_T_x,_ which impeded the formation of the high-valence species required for OER. Therefore, improved conductivity alone cannot confirm a beneficial role of MXene; the interfacial electronic interaction must also permit the generation of the actual working phase [120].

Accordingly, beneficial reconstruction should be assigned only when operando or post-OER characterization confirms the formation of an active oxide/oxyhydroxide phase while the MXene-supported conductive network remains functional. By contrast, increasing content of metallic oxide produced by dissolution of MXene, loss of metal–C signals in MXene phases, rising charge-transfer resistance, active-site blockage, and structural collapse should be regarded as foreshadow of destructive degradation. The results of Operando Raman or XAS should therefore be combined with quasi-in situ XPS, post-OER microscopy, ICP analysis, EIS, Faradaic-efficiency measurements, and quantitative O_2_ detection. This distinction is particularly important because anodic oxidation of MXene itself may contribute parasitic current that cannot be assigned to OER solely from the measured current density.

## 5. Conclusions and Outlook

In summary, the development of MXene-based OER catalysts has evolved from an early stage in which MXenes were mainly regarded as highly conductive two-dimensional supports to a stage of rational design based on multilevel synergistic regulation throughout the entire reaction process. This review has been systematically organized around three core lines, namely vacancy and other atomic-level regulation, interface engineering, morphology engineering and composite engineering. Although these modification strategies differ in regulation scale and mechanistic mode, they ultimately serve the same central objective, namely the synergistic enhancement of OER catalytic performance in MXene-based systems through the regulation of charge transfer, reaction-intermediate adsorption, oxygen-activation pathways, working-state formation and structural stability under high current densities. Vacancy and other atomic-level regulation targets the local coordination environment and electron-density distribution of the catalyst, thereby enabling direct and precise control over the bonding state of active centers, the process of water activation and the formation behavior of high-valent active sites. Interface engineering induces charge redistribution, strong electronic coupling and surface reconstruction through the construction of heterointerfaces, thereby enabling MXenes to move beyond the role of conventional passive conductive frameworks and instead act as key reaction platforms for regulating intermediate adsorption and the formation of active species. Morphology engineering further extends the regulation dimension to interlayer space, intralayer mass-transport channels and open outer-surface architecture, thereby markedly improving active-site utilization under thick-electrode and high current density conditions by optimizing electrolyte wetting, ion diffusion and bubble release. Composite engineering takes the whole multi-component catalyst as the optimization object, and selects different components according to different requirements. Finally, it can overcome the limitations of different components and realize functional complementarity.

The core advantage of MXenes lies not only in their excellent intrinsic conductivity, but more importantly in their highly tunable surface chemistry, designable interlayer space and strong interfacial coupling capability, which enable them to play synergistic roles in electron transport, active-site construction, the regulation of reaction-intermediate adsorption and mass transport, as well as structural stability at high current density. Meanwhile, several key scientific issues in current research on MXene-based OER catalysts still remain to be resolved urgently. The first challenge faced by MXene-based systems is the instability of their initial material state. The composition of surface terminations, defect concentration, interlayer structure and initial degree of oxidation all depend strongly on the etching, delamination, post-treatment and compositing routes. Different preparation pathways often lead to marked differences in the final products, making the actual structures of the catalysts under reaction conditions inconsistent and thereby increasing the difficulty of reproducible validation. Beyond the differences in the initial material state, the actual active sites, working-state evolution pathway and the specific mechanistic role of MXenes in MXene-based OER systems still lack a systematic and conclusive body of experimental evidence. In particular, the causal relationships among the initial MXene, the interfacial active phase, the reconstructed surface species formed during operation and the local coordination environment have not yet been fully clarified. Although some systems exhibit high activity under laboratory conditions, fundamental issues such as MXene-sheet oxidation, structural collapse and interfacial destabilization remain unresolved under complex practical conditions involving high current densities, long-term operation and real electrolyzer devices. This concern is consistent with recent studies on MXene oxidation and stabilization, which show that potential-dependent oxidation, surface chemistry and edge/surface passivation should be considered when evaluating the durability of MXene-based catalytic systems under long-term anodic operation [121,122,123,124,125]. This indicates that current understanding of material durability under realistic working environments is still clearly insufficient.

In the future, research on MXene-based OER catalysts should further shift from the optimization of individual performance metrics toward overall system design oriented to realistic industrial operating conditions. First, a clearer preparation–structure relationship needs to be established around the initial material state, with emphasis on improving the controllability and reproducibility of surface terminations, defect concentration, interlayer structure and degree of oxidation. Second, in situ characterization techniques should be combined with theoretical calculations to systematically track the formation of active sites, the evolution of the working state and the process of interfacial reconstruction, so as to quantitatively clarify the relative contributions of interfacial effects, structural effects and local-site effects. Third, greater emphasis should be placed on tests under high current density, long-term stability evaluation and device-level performance assessment. Thus, a continuous evaluation framework from laboratory conditions to actual operation environment should be established and the structural maintenance ability and long-term operation reliability of the system based on MXene should be truly tested. Ultimately, improving the anodic durability of MXene-based OER catalysts requires stabilization at both the surface and structural levels. Selective passivation of oxidation-prone edges and defects can suppress the initial attack of oxygen while preserving accessible catalytic sites. Thin protective coatings may further isolate the MXene framework from the strongly oxidative environment, but they must remain electronically conductive and permeable to OH^−^ and O_2_. Surface-termination control provides a more intrinsic strategy because the type and coverage of O, OH, F, or halogen terminations could optimize metal–carbon bonding, electronic conductivity, and resistance to oxidation. In addition, choosing more oxidation-resistant MXene compositions and optimizing MXene loading can reduce the accumulation of insulating oxides.

The true breakthrough of MXene-based OER catalysts lies not in the improvement of a single performance parameter, but in the ability to develop a comprehensive design framework that covers the range from the atomic scale to the device scale, from the initial state to the operating state, and from short-term activity to long-term durability. We hope this review can provide theoretical guidance and a roadmap reference for the design and evaluation of efficient and stable MXene-based anode materials in the future.

## Figures and Tables

**Table 1:** Summary of the OER performance of vacancy and other atomic-level-engineered MXene-based catalysts.

Catalyst	Regulation Strategy	Electrolyte	η @ j (mV@mA cm^−2^)	Tafel Slope (mV dec^−1^)	Stability	Faraday Efficiency	Ref.
Ti_3_C_2_T_x_-N_6_	N doping/plasma delamination	1 M KOH	360@10	76.68	12 h@100 mA cm^−2^	—	[34]
V-Co_2_P@HE	V doping + interfacial coupling	1 M KOH	227@10	44.45	48 h@ 100 mA cm^−2^	94.8%	[35]
MXene@Ce-MOF	O-vacancy engineering	1 M KOH	270@10	163.8	12 h@10 mA cm^−2^	—	[41]
Co_9_S_8-x_/Ti_3_C_2_T_x_	S-vacancy engineering	1 M KOH	286@10	76	50 h@100 mA cm^−2^	90.06%	[46]
p-Ti_3_C_2_/TiO_2_-CoMoO_4_-NiNC-NF	O vacancies + partial oxidation	1 M KOH	190@10	56.1	200 h@100 mA cm^−2^	—	[47]
Co@V_2_CT_x_	Co single atoms	1 M KOH	242@10	90.4	10 h@10 mA cm^−2^	—	[48]
CoNi-Ti_3_C_2_T_x_	Co/Ni dual atoms	1 M KOH	241@10	79.8	>100 h@500 mA cm^−2^	—	[49]
t-RuO_2_ atomic layers/Mo_2_TiC_2_T_x_ (RAL-M)	Atomic RuO_2_ layers/defect modulation	O_2_-saturated 0.5 M H_2_SO_4_	222 ± 1@10	50.4	22 h@10 mA cm^−2^	—	[55]
Ir@Ti_3_C_2_T_x_ (working-state IrO_2_/TiO_x_)	Operando oxidation/active-interface formation	0.1 M HClO_4_	295@10	66	>50 h@10 mA cm^−2^	≈100%	[3]

**Table 2:** Summary of the OER performance of interface-engineered MXene-based catalysts.

Catalyst	Electrolyte	η @ j (mV @ mA cm^−2^)	Tafel Slope (mV dec^−1^)	Stability	Ref.
H_2_PO_2_^−^/FeNi-LDH–V_2_C	1 M KOH	250@10	46.5	50,000 s@250 mV	[70]
FeNi-LDH/Ti_3_C_2_-MXene	1 M KOH	298@10	43	12 h@10 mA cm^−2^	[72]
CoNi-LDH/Ti_3_C_2_T_x_/NF	1 M KOH	257.4@100	68	50 h@10 mA cm^−2^	[71]
CoFe-LDH/MXene	1 M KOH	319@10	50	10 h@0.5 V	[73]
HEBO_x_/MXene3	1 M KOH	268@10	39.8	45 h@10 mA cm^−2^	[78]
t-RuO_2_ atomic layers/Mo_2_TiC_2_T_x_ (RAL-M)	O_2_-saturated 0.5 M H_2_SO_4_	222 ± 1@10	50.4	22 h@10 mA cm^−2^	[55]
Ir@Ti_3_C_2_T_x_ (working-state IrO_2_/TiO_x_)	0.1 M HClO_4_	295@10	66	>50 h@10 mA cm^−2^	[3]

**Table 3:** Summary of the OER performance of morphology-engineered MXene-based catalysts.

Catalyst	Electrolyte	η @ j (mV @ mA cm^−2^)	Tafel Slope (mV dec^−1^)	Stability	Faraday Efficiency	Ref.
P-MoO_3_ FCL MXene/NF	1 M KOH	179@10	40.44	1000 CV cycles	96.30%	[90]
Co_4_Fe_1_-LOMX	Alkaline; concentration NR	301@10	43	200 h@10 mA cm^−2^	—	[91]
TS(24)-P(50)-V_2_C	1 M KOH	269@10	85.1	1000 CV cycles	—	[92]
NiFe/MX-HT	O_2_-saturated 1 M KOH	230@10	38	≈220 h@10 mA cm^−2^	≈100%	[93]
LDH(60%)/H–Ti_3_C_2_T_x_	1 M KOH	270@20	47	24 h@100 mA cm^−2^	—	[94]
TS-V_2_CT_x_/CoV_2_O_6_ HN	1 M KOH	235@10	71.8	>24 h@10 mA cm^−2^	—	[95]
ZnCoCH@ Ti_3_C_2_T_x_	1 M KOH	280@10	46.2	1000 CV cycles	≈100%	[96]
CoNi-LDH/MXene@NiMoO_4_	1 M KOH	222@100	84.2	20 h@300 mA cm^−2^	—	[101]
CoFe-P@MXene/NF	1 M KOH	215@20	48.1	100 h@100 mA cm^−2^	>97%	[102]
RuO_2_-Ti_3_C_2_/NF	1 M KOH	351@100	127.5	40 h@20 mA cm^−2^	100%	[106]
Ir@Ti_3_C_2_T_x_ (working-state IrO_2_/TiO_x_)	0.1 M HClO_4_	295@10	66	>50 h@10 mA cm^−2^	≈100%	[3]

**Table 4:** Summary of the OER performance of composite-engineered MXene-based catalysts.

Catalyst	Composite Class	η @ j (mV @ mA cm^−2^)	Tafel Slope (mV dec^−1^)	Stability	Ref.
NiFe-LDH/Ti_3_C_2_T_x_-rGO	LDH/MXene/rGO	235@10	40	12 h@10 mA cm^−2^	[112]
MNG-MoSn_2_Se_4_	Selenide/MXene/rGO	50@10	55.9	10 h@open-circuit potential	[113]
CoF_2_/MXene	Fluoride/MXene	275@10	50.7	2000 CV cycles	[115]
MXene/NiFeP_x_–NC	Phosphide/NC/MXene	240@10	81.2	2000 CV cycles	[114]
1.0-LDH/3MXNF	LDH/MXene/NF	247@100	67.7	24 h@50 mA cm^−2^	[81]
RuO_2_-Ti_3_C_2_/NF	1 M KOH	351@100	127.5	40 h@20 mA cm^−2^	[106]

## Data Availability

No new data were created or analyzed in this study. Data sharing is not applicable to this article.
